# Supplementary material for: WordCommentsAnalyzer: A windows software tool for qualitative research
Source: F1000Res. 2018 Sep 4;7:536. Originally published 2018 May 3. [Version 2] doi: 10.12688/f1000research.14819.2 (PMC6137411; doi:10.12688/f1000research.14819.2)
Supplement: Supplementary file 3 [file f1000research-7-17614-s0002.tgz › b2a79496-4ada-47c4-9682-a99ce867836d.zip!/Eight Iranian Health Journals/Journal of Health System Research _39.docx]

© Journal of Health System Research . This work is licensed under CC BY-NC 4.0 (https://creativecommons.org/licenses/by-nc/4.0/).

Volume & Issue: دوره 7، شماره 6؛1390

Title: مروری بر موازین و دستورالعمل‌های اخلاقی در چرخه پژوهش‌های مرتبط با سلامت

http://hsr.mui.ac.ir/index.php/jhsr/article/view/395

چرخه پژوهش با پرسش یک سؤال و یا طرح یک فرضیه آغاز می‌گردد و تا یافتن پاسخ آن سؤال یا آزمون آن فرضیه ادامه یافته، نتایج حاصل از هر پژوهش زمانی به سرانجام می‌رسد که در قالب یک مقاله پژوهشی به رشته تحریر در آمده و در یک نشریه معتبر به چاپ برسد. در طی هر یک از این مراحل، پژوهشگران نیازمند رعایت نکات و دستورالعمل‌های مرتبط با اخلاق در پژوهش می‌باشند. این نکات اخلاقی از هنگام طرح سؤال یا تدوین فرضیه آغاز شده، تا هنگام نوشتن و اجرای طرح پژوهشی ادامه می‌یابند. آن‌ها همچنین، در انجام تجزیه و تحلیل داده‌ها و نوشتن و انتشار دستاوردهای پژوهشی نیز مطرح می‌گردند. این نكات آن قدر حایز اهمیت هستند كه تنها پژوهش‌هایی كه تمامی نکات اخلاقی را مورد نظر قرار داده باشند نکته جدیدی را به دانش موجود اضافه خواهند نمود. هدف از نگارش مقاله حاضر، مروری بر مهم‌ترین نكات و دستورالعمل‌های اخلاقی در چرخه پژوهش‌های مرتبط با سلامت، با بهره‌گیری از منابع موجود می‌باشد.

Title: جایگاه حمایت اجتماعی در خودمراقبتی بیماران مبتلا به دیابت؛ مطالعه مروری

http://hsr.mui.ac.ir/index.php/jhsr/article/view/409

مقدمه: دیابت یکی از نگرانی‌های عمده هزاره سوم میلادی به شمار می‌رود که روز به روز بر شمار مبتلایان به این بیماری افزوده می‌شود. وضعیت این بیماری در کشورمان نیز نشان می‌دهد که شیوع بیماری بالا و حدود 7/7 درصد است. مهم‌ترین راهکار کنترل و پیشگیری از عوارض دیابت، خودمراقبتی می‌باشد که بر اساس مطالعات متعدد خودمراقبتی به علل مختلف در بیماران مبتلا به دیابت در شرایط مطلوبی قرار ندارد. این مطالعه به تعیین ارتباط حمایت اجتماعی به ویژه حمایت خانواده بر رفتارهای خودمراقبتی در بیماران دیابتی پرداخته است. روش بررسی: این مطالعه، مروری (Narrative Review) بود و با استفاده از بانک‌های اطلاعاتی و موتورهای جست‌وجوی علمی نظیر PubMed، ProQuest، SCOPUS و Elsevier، و کلید واژگان دیابت، حمایت اجتماعی و خودمراقبتی مقالات با طراحی‌های مقطعی، کوهورت، کارآزمایی بالینی و نیز مرور سیستماتیک انتخاب شدند. همچنین مقالات فارسی با استفاده از بانک‌های اطلاعاتی IRANMEDEX، MEDLIB و نیز جست‌وجوی مقالات از سایت‌های مجلات علمی پژوهشی داخلی انتخاب شدند. بازه زمانی مقالات مورد استفاده از سال 1990 تا پایان سال 2011 میلادی بود. یافته‌ها: نتایج مطالعات بررسی شده حاکی از آن بود که وضعیت خودمراقبتی و نیز حمایت اجتماعی در بیماران دیابتی در حد مطلوبی قرار نداشت. مطالعات مورد بررسی نشان دادند که نه تنها بین حمایت اجتماعی و رفتارهای خودمراقبتی همبستگی مثبتی وجود داشت، بلکه برخی مطالعات حاکی از اثر مثبت حمایت اجتماعی به ویژه حمایت خانواده خاصه همسر بیمار بر کنترل قند خون و HbA1c بود. نتیجه‌گیری: همانطور که حمایت اجتماعی می‌تواند پیش‌بینی کننده رفتار ارتقا دهنده سلامت باشد، این سازه توانایی پیش‌بینی رفتارهای خودمراقبتی در بیماران دیابتی را نیز دارا می‌باشد. از این‌رو، درگیر نمودن اعضای خانواده به خصوص همسر بیمار در فرایند خودمراقبتی، می‌تواند برای ارایه دهندگان خدمات سلامی به بیماران دیابتی حایز اهمیت باشد.

Title: تبیین نقش خودکارآمدی (Self-efficacy) در خودمراقبتی بیماران دیابتی

http://hsr.mui.ac.ir/index.php/jhsr/article/view/446

مقدمه: بيماري ديابت از جمله بيماري‌هاي مزمن و به نسبت شايع در جهان است که ظرف 20 سال گذشته تعداد مبتلايان به آن در دنيا هفت برابر شده است و در صورت تداوم روند فعلي، با افزايش سالانه 7 ميليون نفر به جمعيت اين بيماران، تا سال 2030، اين تعداد به 350 ميليون نفر مي‌رسد. تا کنون عوامل غیر قابل تعدیل مختلفی در خودمراقبتی این بیماری جهت کنترل بیماری و پیشگیری از عوارض زودرس و دیررس آن بررسی شده است. این مطالعه سعی داشت نقش خودکارآمدی را به عنوان عاملی قابل تعدیل در خودمراقبتی بیماران دیابتی تبیین نماید. روش بررسی: این مطالعه، مروری (Narrative Review) بود. با استفاده از بانک‌های اطلاعاتی و موتورهای جستجوی علمی نظیر PubMed، ProQuest، SCOPUS و Elsevier و واژه‌های کلیدی «دیابت»، «خودمراقبتی» و «خودکارآمدی»، مقالات با طراحی‌های مقطعی، کوهورت، کارآزمایی بالینی و نیز مرور سیستماتیک انتخاب شدند. همچنین مقالات فارسی با استفاده از بانک‌های اطلاعاتی IRANMEDEX، MEDLIB و نیز جست‌وجو در سایت‌های مجلات علمی- پژوهشی داخلی انتخاب شدند. بازه زمانی مقالات مورد استفاده از سال 1990 تا پایان سال 2011 میلادی بود. نتایج: نه تنها شرایط خودمراقبتی در بیماران دیابتیک مطلوب نمی‌باشد، بلکه نتایج مطالعات بررسی شده حاکی از آن بود که وضعیت خودکارآمدی نیز در این بیماران پایین است. همچنین یافته‌های مطالعات بررسی شده نشان دادند که بین خودکارآمدی و خودمراقبتی در این بیماران همبستگی مثبتی وجود دارد، به طوری که این سازه می‌تواند قابلیت پیش‌بینی کنندگی رفتار مراقبت از خود را داشته باشد. نتیجه‌گیری: در روند کنترل بیماری دیابت، خودمراقبتی اهمیت فراوان دارد. خودکارآمدی به طور مستقیم از طریق انتظارات کارآمدی، موجب انگیزه برای رفتار ارتقا دهنده سلامتی می‌شود، همچنین به طور غیر مستقیم از طریق موانع درک شده و تعیین میزان تعهد یا ثبات قدم برای ادامه پیگیری نقشه عمل، انگیزه را تحت تأثیر قرار می‌دهد. بنابراین در فرایند تغییر رفتار خودمراقبتی، ارتقای خودکارآمدی بسیار حایز اهمیت است.

Title: بررسی نقش منافع و موانع درک شده در انجام رفتارهای خودمراقبتی در بیماران دیابتیک

http://hsr.mui.ac.ir/index.php/jhsr/article/view/448

مقدمه: در بیماری‌ها و اختلالاتی نظیر دیابت، درمان و مدیریت بیماری به طور عمده به اقدامات بیمار وابسته است. ازاین‌رو، خودمراقبتی در این بیماران از اهمیت به سزایی برخوردار است و بیماران با کسب مهارت‌های مراقبت از خود می‌توانند بر آسایش، توانایی‌های عملکردی و فرایندهای بیماری تأثیرگذار باشند. امروزه می‌دانیم خودمراقبتی تابعی از عوامل فردی، روانی و اجتماعی است که شناخت و درک آن می‌تواند سیستم‌های ارایه دهنده خدمات درمانی را جهت اجرای برنامه‌های آموزشی یاری دهد. این مطالعه با هدف بررسی نقش منافع و موانع درک شده در انجام رفتارهای خودمراقبتی در بیماران مبتلا به دیابت صورت گرفت. روش بررسی: این مطالعه، یک مطالعه مروری (Narrative Review) بود و با استفاده از بانک‌های اطلاعاتی و موتورهای جست‌وجوی علمی نظیر PubMed، ProQuest، SCOPUS، Elsevier و واژه‌های کلیدی «خودمراقبتی»، «دیابت»، «منافع (فواید) درک شده» و «موانع درک شده» مقالات با طراحی‌های مقطعی، کوهورت، مداخله‌ای،کارآزمایی بالینی، و نیز کیفی و مروری انتخاب شدند. همچنین مقالات فارسی با استفاده از بانک‌های اطلاعاتی IRANMEDEX، MEDLIB و نیز جست‌وجوی مقالات از سایت‌های مجلات علمی- پژوهشی داخلی انتخاب شدند. نتایج: یافته‌های مطالعات بررسی شده حاکی از وضعیت متوسط منافع و موانع درک شده در بیماران دیابتی در خصوص خودمراقبتی بود. از مهم‌ترین منافع درک شده می‌توان به کنترل بهتر قند خون، کنترل وزن بدن، احساس شادابی و کاهش هزینه‌ها اشاره کرد. همچنین مهم‌ترین موانع درک شده در مراقبت از خود عدم حمایت خانواده، احساس خجالت، فراموشی و عدم چشم‌پوشی از طعم غذاها بود. نتیجه‌گیری: منافع و موانع درک شده به عنوان سازه‌های مرکزی در برخی الگوها و نظریات تغییر رفتار با خودمراقبتی مرتبط بود، به طوری که خودمراقبتی با منافع درک شده ارتباط مستقیم و با موانع درک شده ارتباط معکوس داشت. به همبن دلیل این دو سازه می‌توانند به عنوان استراتژی‌هایی جهت ارتقای رفتارهای مراقبت از خود در بیماران دیابتیک مورد توجه قرار گیرند.

Title: کاربرد سيستم‌هاي وتلند ساختگي با جريان زيرسطحي در تصفيه فاضلاب کشورهاي خارجي و ايران

http://hsr.mui.ac.ir/index.php/jhsr/article/view/440

مقدمه: انتخاب روش‌هاي گوناکون تصفیه به عوامل مختلفي همچون نوع فاضلاب، موقعيت جغرافيايي، کميت و کيفيت فاضلاب، کيفيت پساب خروجي، فناوري‌ها، امکانات موجود، بودجه و ... بستگي دارد. روش‌هاي طبيعي تصفيه از جمله وتلندها براي کشورهاي در حال توسعه به خصوص شهرهای کوچک و دور افتاده ساده، ارزان و قابل قبول مي‌باشند. وتلندها شامل دو دسته طبيعي و ساختگي هستند. وتلندهاي ساختگي نيز شامل دو نوع وتلند ساختگي با جريان زيرسطحي و وتلند ساختگي با جريان روسطحي می‌باشند. هدف اين مقاله، بررسي وضعيت و سابقه استفاده از وتلند در ايران و ساير کشورها بود. روش‌ها: مطالعه از نوع مروري بوده است که با هدف بررسي کارهاي انجام شده و مقايسه کارايي سيستم‌هاي وتلند با جريان زيرسطحي در تصفيه فاضلاب ايران و کشورهاي خارجي انجام شده است. یافته‌ها: ميانگين درصد حذف در تصفيه فاضلاب با وتلندهاي زيرسطحي در کشورهاي خارجي براي اکسيژن مورد نياز بيوشيميايي (Biochemical oxygen demand یا BOD)، کل جامدات معلق (Total suspended solids یا TSS)، فسفر کل (Total phosphorus یا TP) و نيتروژن کل (Total nitrogen یا TN) به ترتيب در حدود 6/91، 6/81، 8/78 و 8/70 درصد و ميانگين راندمان حذف در 6 کار عملي انجام شده در ايران برای BOD، TSS، TP و TN نيز به ترتيب در حدود 77،83، 78 و 83 درصد بود. نتيجه‌گيري: نتايج تحقيقات در کشورهاي خارجي نشان داده است که وتلند براي انواع مختلفي از فاضلاب‌ها کاربرد دارد و راندمان مناسبي را در تصفيه انواع فاضلاب‌ها برآورده مي‌سازد. متأسفانه در ايران استفاده از وتلند جهت تصفيه انواع مختلف فاضلاب جايگاه خاصي ندارد و تنها چند کار عملي محدود که آن هم مربوط به فاضلاب خانگي است، صورت گرفته است.

Title: بررسی عوامل پیش‌بینی کننده قصد رفتار ایمن کارگران با استفاده از الگوی رفتار سالم

http://hsr.mui.ac.ir/index.php/jhsr/article/view/382

مقدمه نگرانی جهان شمول در زمینه حوادث کاری و ایمنی، نیاز به شیوه‌های جدید و مؤثر برای بهبود ایمنی در محل کار را به طور کامل محسوس کرده است. برای افزایش کارایی مداخلات پیش‌گیرانه لازم است به تعیین کننده‌های رفتار پرداخته شود. قصد رفتاری، یکی از تعیین کننده‌های اصلی رفتار است. هدف از این مطالعه تعیین عوامل پیش‌بینی کننده قصد رفتار ایمن کارگران با استفاده از الگوی رفتار سالم بود.   مواد و روش این مطالعه توصیفی در سال 1388 انجام گرفت و الگوی رفتار سالم (HAM) را در کارگران دو بخش مهندسی نورد و فولادسازی ذوب‌آهن اصفهان جهت پیش‌بینی قصد رفتار ایمن به کار برد. به روش نمونه‌گیری تصادفی تعداد 300 نفر از کارگران این دو قسمت انتخاب شدند. داده‌ها توسط پرسش‌نامه محقق ساخته بر اساس الگوی رفتار سالم جمع‌آوری شد و به کمک تحلیل عاملی اکتشافی و رگرسیون چندگانه با نرم‌افزار 13SPSS مورد تجزیه و تحلیل قرار گرفت. یافته‌ها میانگین و انحراف معیار سن کارگران 38/7 ± 6/34 و میانگین و انحراف معیار سابقه کار آنان 71/7 ± 7/11 سال بود. سه عامل سیستم اعتقادی، سیستم نگرشی و سیستم هنجاری، 25 درصد از واریانس قصد رفتار ایمن در کارگران را تبیین کردند (001/0 < P). نتیجه‌گیری نتایج مطالعه از الگوی رفتار سالم به عنوان الگویی مؤثر برای توضیح قصد رفتاری حمایت کردند. این الگو می‌تواند به عنوان چهارچوبی برای طراحی مداخلات ایمنی سودمند مورد استفاده قرار گیرد.

Title: بررسی نگرش جوانان در شرف ازدواج در رابطه با شناخت همسر آینده و زندگی مشترک در شهرستان بروجن

http://hsr.mui.ac.ir/index.php/jhsr/article/view/383

مقدمه: ازدواج را می‌توان یکی از مراحل مهم سه گانه زندگی (تولد، ازدواج و مرگ) به حساب آورد. در صورتی این مرحله بدون دغدغه طی می‌شود که شروع آن مناسب بوده، دختر و پسر نسبت به یکدیگر شناخت کافی داشته باشند، زیرا عدم شناخت آن‌ها نسبت به یکدیگر موجب از هم پاشیدگی و جدایی در خانواده می‌شود. از این رو مطالعه حاضر با هدف بررسی نگرش جوانان در شرف ازدواج در رابطه با شناخت همسر آینده و زندگی مشترک آن‌ها صورت گرفت. روش ها: پژوهش حاضر از نوع مقطعی (توصیفی- تحلیلی) بود که در شهرستان بروجن در مرکز مشاوره قبل از ازدواج بر روی 200 نفر از زوجین در شرف ازدواج در سال 1389 با استفاده از یک پرسش‌نامه محقق ساخته انجام گردید که روایی و پایایی آن بررسی شده بود. این پرسش‌نامه شامل بر سه بخش اطلاعات دمگرافیک، سؤالات زمینه‌ای در ارتباط با زمان و نحوه آشنایی زوجین و گویه‌های نگرشی بوده است. امتیاز کامل نگرش این پرسش‌نامه 68 نمره بوده است. اطلاعات حاصل از این پرسش‌نامه در نرم‌افزار SPSS نسخه 18 وارد شده، در نهایت مورد تجزیه و تحلیل قرار گرفت. یافته‌ها: میانگین نمره نگرش پسران 27/5 ± 3/43 و دختران 42/5 ± 87/41 بوده، که حداقل و حداکثر نمره به دست آمده در دو جنس به ترتیب برابر با 23 و 57 بوده است. همچنین ارتباط آماری معنی‌داری بین جنسیت و قبول همسر آینده با شرایط موجود (009/0 = P)، توانمند بودن برای زندگی مشترک آینده (001/0 > P)، اطمینان کافی برای ازدواج (001/0 > P)، اعتقاد به فایق شدن بر مشکلات زندگی آینده (009/0 = P) وجود داشته؛ به طوری که در همه موارد فوق دختران نگرش ضعیف‌تری از خود، شوهر و زندگی خود داشته‌اند. در این مطالعه 34 درصد از زوجین با همسر آینده خود خویشاوند و 5/25 درصد زوجین تحصیلات دانشگاهی داشته‌اند. نتیجه‌گیری: به طور کلی نگرش دختران و پسران در رابطه با شناخت همسر آینده و زندگی مشترک در سطح متوسطی بوده، برگزاری کلاس‌های مشاوره ازدواج و مهارت‌های زندگی برای زوجین و شناخت بیشتر همسر به خصوص برای دختران، قبل از ازدواج ضروری به نظر می‌رسد.

Title: "بررسی میزان رضایت شغلی کارکنان شبکه بهداشتی درمانی خمینی شهر در سال 1389"

http://hsr.mui.ac.ir/index.php/jhsr/article/view/384

مقدمه: سازمان‌های بهداشتی به علت اهمیت وظیفه‌ای که در زمینه پیش‌گیری و مراقبت به عهده دارند، از جایگاه ویژه‌ای برخوردارند. نارضایتی شغلی در بین پرسنل این سازمان‌ها منجر به گسستگی عاطفی، بی‌تفاوتی و کاهش کیفیت خدمات ارایه شده می‌شود، که این امر نتایج جدی را در برخواهد داشت. از این رو این مطالعه با هدف بررسی رضایت شغلی در پرسنل بهداشتی انجام شد. روش‌ها: این مطالعه توصیفی- تحلیلی از نوع مقطعی بود كه کارکنان بهداشتی تحت پوشش شبکه بهداشت و درمان خمینی‌شهر (مراکز، پایگاه‌ها، خانه‌ها و شبکه) در صورت داشتن حداقل 6 ماه سابقه کار، وارد مطالعه شدند. تعداد افراد مورد مطالعه 197 نفر بودند. ابزار گردآوري داده‌ها، پرسش‌نامه دو قسمتی شامل ویژگی‌های فردی به همراه پرسش‌نامه استاندارد توصیف شغلی Smith (JDI) بود که این پرسش‌نامه دارای 6 حیطه می‌باشد. پایایی برای هر حیطه به دست آمد و روایی پرسش‌نامه نیز 93/0گزارش شده است. پس از جمع‌آوري داده‌ها، اطلاعات از طریق نرم‌افزار SPSS وارد کامپیوتر شده، با استفاده از آزمون‌های آماری همبستگی Pearson، همبستگی Spreaman، آنالیز واریانس و t مستقل مورد تجزیه و تحلیل قرار گرفت و نتایج اعلام شد. يافته‌ها: میانگین نمره رضایت شغلی کلی 8/12 ± 55/43 (از 100) به دست آمد. اگر چه نمره رضایت شغلی بین دو جنس تفاوت معنی‌داری نداشت. در حیطه نگرش به شغل فعلی (ماهیت شغل)، رضایت مردان بیشتر از زنان بود (001/0 = P). بیشترین نمره رضایت شغلی در حیطه روابط با همکاران و کمترین نمره رضایت شغلی در حیطه حقوق و مزایا و ارتقای شغلی به دست آمد. بین نمره رضایت شغلی در حیطه سرپرست با سابقه کار رابطه معکوس و بین نگرش به شغل فعلی با درآمد رابطه مستقیم به دست آمد (01/0 = P). ارتباط معنی‌دار معکوسی بین سطح تحصیلات فرد و رضایت در حیطه ارتقای شغلی (01/0 = P، 17/0- = r) و حقوق و مزایا (01/0 = P، 22/0- = r) وجود داشت. به عبارتی افراد با تحصیلات بالاتر از میزان حقوق و شرایط ارتقای خود رضایت کمتری داشتند.   بحث و نتيجه‌گيري: به دلیل آن که رضایت شغلی، بر تعهد کارکنان و میزان جابه‌جایی و غیبت آنان تأثیر به سزایی دارد، بر مدیران لازم است که ضمن توجه به ابعاد رضایت شغلی به حل و فصل مشکلات پرداخته، تا رضایت شغلی و در نتیجه کیفیت کار افزایش یابد.

Title: بررسي فاکتورهای مؤثر بر انجام فعالیت فیزیکی منظم در بین پرسنل فوریت‌های پزشکی استان همدان با بهره‌گیری از مدل اعتقاد بهداشتی

http://hsr.mui.ac.ir/index.php/jhsr/article/view/385

مقدمه: در حال حاضر با توجه به ماشینی شدن شیوه زندگی و کاهش تحرک و فعالیت فیزیکی بیماری‌های غیر واگیر در حال افزایش است. در این بین پرستاران و پرسنل فوریت‌های پزشکی از جمله افرادی هستند که به واسطه شغل پراسترس خود و شیفت‌های کاری فشرده فعالیت فیزیکی کافی ندارند. این مطالعه با هدف بررسي فاکتورهای مؤثر بر فعالیت فیزیکی منظم در بین پرسنل فوریت‌های پزشکی استان همدان با بهره‌گیری از مدل اعتقاد بهداشتی انجام شد. روش‌ها: این پژوهش یک مطالعه توصیفی- تحلیلی بود که بر روي پرسنل فوريت‌هاي پزشكي استان همدان انجام گرفت. نحوه انجام مطالعه بدين شرح بود كه ابتدا كليه پرسنل استخدامی رسمی و پیمانی پايگاه‌هاي اور‍ژانس استان همدان براي مطالعه انتخاب شدند که تعداد آنان 102 نفر بود. سپس پرسش‌نامه طراحی شده بر مبنای مدل اعتقاد بهداشتي و پرسش‌نامه فعالیت فیزیکی (IPAQ) در اختیار آنان قرار داده شد. کلیه تجزیه و تحلیل‌های این پژوهش به وسیله ورود داده‌ها به برنامه SPSS انجام گرفت. يافته‌ها: در این مطالعه 5/73 درصد از افراد فعالیت جسمانی ضعیف داشتند. همچنین نتایج نشان داد که بین خودکارامدی ورزش و فعالیت فیزیکی منظم رابطه معنی‌دار آماری وجود دارد (036/0 = P). بین فواید و موانع درک شده با خودکارامدی ورزش نیز رابطه معنی‌دار آماری وجود دارد (001/0 = P). اما بین فواید و موانع درک شده با انجام فعالیت فیزیکی رابطه معنی‌داری یافت نشد. نتیجه‌گیری: با توجه به یافته‌های پژوهش، بیشتر پرسنل فعالیت جسمانی کافی ندارند، از این رو با تقویت خودکارامدی ورزش و از طرفی ارتقای آگاهی و دانش آنان در مورد فواید و موانع ورزش باید زمینه انجام فعالیت فیزیکی منظم را به ویژه در محیط کار افزایش داد.

Title: ارزیابی و کنترل آلودگی صدای مولدهای دیزلی برق جوشکاری در کارگاه‌های ساختمان‌سازی شهر همدان

http://hsr.mui.ac.ir/index.php/jhsr/article/view/386

مقدمه: مولدهای دیزلی که جهت تأمین برق مورد نیاز جوشکاری مورد استفاده قرار می‌گیرند، می‌توانند سبب آلودگی صوتی در محدوده کارگاه‌های ساختمان‌سازی شوند. مطالعه حاضر با هدف ارزیابی صدای این دستگاه‌ها و ارایه راه‌کار کنترلی مناسب در شهر همدان صورت پذیرفته است.  روش‌ها: در این مطالعه توصیفی- تحلیلی و تجربی، تراز فشار صوت در فاصله یک متری و در 4 نقطه اطراف دستگاه‌ها و در مجاورت معابر اندازه‌گیری گردید و برای ایستگاه‌های بالاتر از حد مجاز آنالیز فرکانس نیز صورت گرفت. سپس با استفاده از پرسش‌نامه طراحی شده، آزار صوتی همسایگان مجاور کارگاه‌ها مورد بررسی قرار گرفت. جهت کاهش تراز فشار صوت این مولدها متناسب با اهداف مطالعه و محاسبات طراحی از انباره مناسب موجود در بازار استفاده گردید و داده‌ها با استفاده از SPSS نسخه 13 مورد تحلیل قرار گرفت. یافته‌ها: میانگین و انحراف معیار SPL در اطراف دستگاه‌ها (A) dB2/2 ± 1/97 و میانگین و انحراف معیار آن در معابر اطراف آن برابر با (A) dB4/7 ± 0/74 بوده، که هر 2 مورد بالاتر از حد مجاز تعیین شده بود. 7/60 درصد از همسایگان آزار صوتی ناشی از صدای فعالیت‌ها را بالا می‌دانستند. با استفاده از انباره انتخاب شده، کاهش تراز فشار صوتی در مدل بنز برابر با 4/24 دسی‌بل در فرکانس 63 هرتز و 11 ‌دسی‌بل در تراز فشار کلی صوت و در مدل رومانی به ترتیب 2/21 و 5/9 دسی‌بل حاصل گردید. نتیجه‌گیری: صدای اگزوز عامل اصلی صدای این دستگاه‌ها است، که با انباره مناسب می‌توان به کاهش تراز فشار صوت قابل قبولی در فرکانس اصلی دست یافت.

Title: بررسی دانش، نگرش و عملکرد دانشجویان پزشکی و دندان‌پزشکی دانشگاه علوم پزشکی رفسنجان پیرامون پژوهش در سال 1388

http://hsr.mui.ac.ir/index.php/jhsr/article/view/387

مقدمه: در نظام آموزشی، نیاز گسترده‌ای به تربیت نیروی انسانی پژوهشگر وجود دارد و اهمیت پژوهش در توسعه کشورها و دست‌یابی به استقلال و رشد در تمامی زمینه‌ها امری انکارناپذیر است. در این مطالعه، نگرش، دانش و عملکرد دانشجویان پزشکی و دندان‌پزشکی دانشگاه علوم پزشکی رفسنجان پیرامون پژوهش بررسی شده است.   روش‌ها: در اين مطالعه توصیفی، 400 نفر از دانشجویان پزشکی و دندان‌پزشکی دانشگاه علوم پزشکی رفسنجان مورد بررسی قرار گرفتند. ابزار جمع‌آوری اطلاعات، پرسش‌نامه پژوهشگر ساخته‌ای مشتمل بر 16 سؤال مربوط به نگرش، 9 سؤال مربوط به دانش، 8 سؤال مربوط به عملکرد بود. اطلاعات پس از جمع‌آوری، با استفاده از نرم‌افزار SPSS و آزمون‌های آماری Fisher و 2χ مورد تجزيه و تحليل قرار گرفتند.   یافته‌ها: بر اساس نتایج، 237 نفر (8/87 درصد) از دانشجویان پزشکی و 100 نفر (9/76 درصد) از دانشجویان دندان‌پزشکی به پرسش‌ها پاسخ دادند. دانش دانشجویان در سطح متوسط و قابل قبول ارزیابی شد. اکثریت آن‌ها نگرش خوبی به پژوهش داشتند. عملکرد پژوهشی دانشجویان در حد متوسط پایین ارزیابی شد. دانشجویان پزشکی نسبت به دندان‌پزشکی از دانش بیشتری برخوردار بودند. دانشجویان پزشکی، سنوات تحصیلی بالاتر، افراد مجرد و افراد ساکن در خوابگاه نگرش بهتری به پژوهش داشتند؛ در حالی که دانشجویان دختر و دانشجویان دندان‌پزشکی از عملکرد پژوهشی بهتری برخوردار بودند.   نتیجه‌گیری: این تحقیق نشان داد که دانشجویان دانشگاه علوم پزشکی رفسنجان اهمیت تحقیقات دانشجویی را درک کرده‌اند و به آن علاقه‌مند هستند، ولی اطلاعات کاملی از ماهیت آن ندارند و به طور عملی نیز با آن در تماس نبوده‌اند. از این رو توصیه می‌شود که جهت ارتقای سطح دانش و بهبود عملکرد آن‌ها برنامه‌ریزی لازم انجام گیرد.

Title: بررسي کيفيت شیمیایی آب شرب روستايي دشت بيرجند و قائن در سال 1389-1388

http://hsr.mui.ac.ir/index.php/jhsr/article/view/389

مقدمه: بالا بودن غلظت املاح در آب از جمله عوامل آلاينده آب‌هاي زيرزميني محسوب مي‌شود که در سال‌هاي اخير به لحاظ افزايش جمعيت و افزايش توليد فاضلاب و افت سطح آب‌هاي زيزميني رو به افزايش گذاشته است. اگر غلظت املاح در آب نوشيدني بيش از حد مجاز باشد، از نظر بهداشتي مخاطره‌آميز مي‌شود. هدف از اين پژوهش اندازه‌گيري پارامترهاي فيزيکي شيميايي آب شرب روستايي دشت بيرجند و قائن و مقايسه آن با استانداردهاي ملي و بين المللي بود. روش‌ها: اين تحقيق به روش توصيفي- مقطعي در پاييز 1388 و بهار 1389 انجام گرفت. در طول مدت تحقيق به طور تصادفي، تعداد 54 نمونه از 27 ايستگاه (27 نمونه در فصل پاييز و 27 نمونه در فصل بهار) برداشت شد و غلظت پارامترهاي فيزيکي- شيميايي اندازه‌گيري شد. در پايان نتايج حاصله با روش آماري توصيفي و دياگرام Shuler مورد بررسي و تجزيه تحليل قرار گرفت. یافته‌ها: مطابق نتايج حاصل از آناليز نمونه‌ها و مقايسه آن‌ها با استاندارد مربوطه چنين برآورد شد که باقيمانده جامدات خشک در 37 درصد از نمونه‌ها، سختي 25 درصد، سولفات 33 درصد، سديم 70 درصد، کلريد 25 درصد، هدايت الکتريکي 51 درصد بيشتر از حداکثر مجاز استاندارد ملي و فلوئور در 92 درصد از نمونه‌هاي آناليز شده کمتر از حداقل فلوئور توصيه شده به دست آمد، همچنين بر اساس دياگرام Shuler پارامترهاي کيفي آب در وضعيت خوب تا نامناسب قرار گرفتند. نتيجه‌گيري: با توجه به خشک‌سالي‌هاي اخير و تأثیر آن بر کيفيت آب‌هاي شرب روستايي و همچنين عدم مطابقت مقادير باقيمانده جامدات خشک، سختي، سولفات، سديم، کلريد، هدايت الکتريکي و فلورايد با استانداردهاي مربوطه، برنامه‌ريزي جهت پایش مستمر منابع آب ضروري به نظر مي‌رسد.

Title: شناسایی و مدل‌سازی الگوهای مرتبط با متاستاز سرطان پستان زنان مراجعه‌کننده به مرکز سید الشهدا اصفهان طی سال‌های 1378 الی 1387 با استفاده از قوانین همبستگی و رگرسیون لجیت رتبه‌ای

http://hsr.mui.ac.ir/index.php/jhsr/article/view/388

مقدمه: سرطان‌ها از بیماری‌های مزمنی هستند که علت‌های ممکن بسیاری دارند. یکی از شایع‌ترین انواع سرطان‌ها، سرطان پستان است که تهدید بزرگی برای سلامتی زنان است. از مسایل مهم در انواع سرطان، انتشار سلول سرطانی به بافت‌های دیگر (متاستاز) است. هدف از مطالعه حاضر بررسی و شناسایی عوامل تأثیرگذار در پیش‌بینی متاستاز سرطان پستان با استفاده از ابزارهای داده‌کاوی می‌باشد. روش‌ها: مطالعه حاضر یک مطالعه مقطعی است که افراد مورد مطالعه (1074 نفر)، بیماران مبتلا به سرطان پستان بودند که طی سال‌های 78 الی 87 به درمانگاه آنکولوژی بیمارستان سید الشهدا اصفهان مراجعه کرده‌اند و تحت درمان قرار گرفته‌اند. تجزیه و تحلیل داده‌ها در حوزه فرایندهای داده‌کاوی و با استفاده از قوانین همبستگی و رگرسیون لجیت رتبه‌ای انجام گرفته است. یافته‌ها: با در نظر گرفتن بیماران با گیرنده‌های هورمونی استروژن و پروژسترون و ژن 53P مثبت، شانس متاستازهای بیشتر در بیماران با نسبت بالای غدد لنفاوی درگیر در زیر بغل، به ترتیب 77/4، 4/1 و 45/4 برابر بیماران با نسبت غدد لنفاوی درگیر کمتراست. در مورد بیماران با وضعیت منفی استروژن، پروژسترون و 53P شانس متاستازهای بیشتر در بیماران با نسبت بالای غدد لنفاوی درگیر در زیر بغل، 97 درصد افزایش دارد. شانس این که بیماران با گیرنده استروژن و ژن 53P مثبت که اندازه تومور آن‌ها بالای 2 سانتی‌متر است، متاستازهای بیشتری داشته باشند، به ترتیب 35/1 و 27/1 برابر بیماران با اندازه تومور کوچک‌تر از 2 سانتی‌متر است. در مورد بیماران با وضعیت منفی نسبت بخت برآورده شده برابر 75/2 بود. نتیجه‌گیری: مطالعه حاضر به معرفی عوامل خطر در ارتباط با انتشار سرطان پستان پرداخته است. عوامل ارایه شده در این مطالعه می‌توانند به شناسایی مجموعه‌ای از بیماران که شانس بیشتری برای ابتلا به متاستاز دارند، کمک کند. همچنین با شناسایی فرایند متاستاز و عوامل مرتبط با آن به بهبود کیفیت زندگی و بقای طولانی مدت بیماران کمک شایانی خواهد شد.

Title: بررسی دانش و نگرش دختران و پسران در شرف ازدواج شهر یاسوج در خصوص بهداشت باروری

http://hsr.mui.ac.ir/index.php/jhsr/article/view/390

مقدمه: امروزه بهداشت باروري به عنوان بخشي از حقوق بشر و با نام حقوق باروري مورد تأييد همگان قرار گرفته است. تدارک بهداشت باروری در سطح ملی و حتی بین‌امللی یکی از گام‌های اساسی در تأمین سلامت جامعه و خانواده می‌باشد. این مطالعه با هدف تعیین آگاهی و نگرش دختران و پسران در شرف ازدواج شهر یاسوج در زمینه بهداشت باروری انجام شد. روش‌ها: این مطالعه توصیفی تحلیلی در سال 1388 در شهر یاسوج انجام گرفت. با روش نمونه‌گیری در دسترس تعداد 777 نفر از داوطلبان ازدواج مراجعه کننده به مرکز بهداشتی درمانی شهید اشرفی شهرستان بویر احمد؛ تنها مرکز مشاوره قبل از ازدواج این شهرستان انتخاب شدند. اطلاعات با پرسش‌نامه محقق ساخته شامل اطلاعات جمعیت شناختی (سن، تحصیلات و شغل) و 10 سؤال چهار گزینه‌ای جهت سنجش آگاهی و 10 سؤال نگرشی در زمینه بهداشت و مراقبت‌های دوران باروری جمع‌آوری شد. اعتبار محتوی پرسش‌نامه با استفاده از نظرات متخصصین پایایی آن با استفاده از آزمون Cronbach's alpha مورد بررسی قرار گرفت. اطلاعات با نرم‌افزار SPSS نسخه 18 مورد تجزیه و تحلیل قرار گرفت. یافته‌ها: میانگین و انحراف معیار سن در دختران 6/4 ± 2/22 سال و در پسران 3/4 ± 36/25 بود. میانگین و انحراف معیار نمره آگاهی و نگرش دختران به ترتیب4/2 ± 6/4 و 6/4 ± 5/10 و میانگین و انحراف معیار نمره آگاهی و نگرش پسران به ترتیب 8/1 ± 47/2 و 7/1 ± 35/3 بود. میانگین نمرات آگاهی و نگرش پسران در مقایسه با دختران مطلوب نبود. همچنین آگاهی و نگرش افراد با تحصیلات بالاتر به طور معنی‌داری بیشتر از افراد با تحصیلات پایین‌تر بود (001/0 ≤ P). بحث و نتیجه‌گیریاجرای برنامه‌های آموزشی مؤثر درباره بهداشت باروری ضروری است. با توجه به نقش مردان در حمایت از اقدامات بهداشتی در برنامه‌های آموزشی پسران باید مورد توجه بیشتری قرار گیرند.

Title: بررسی عوامل روانی- شناختی مرتبط با رفتارهای غربال‌گری سرطان پستان بر حسب مراحل تغییر در معلمین زن شهر اصفهان

http://hsr.mui.ac.ir/index.php/jhsr/article/view/391

مقدمه  سرطان پستان، شایع‌ترین سرطان و دومین علت مرگ و میر ناشی از سرطان در بین زنان است كه در صورت تشخیص در مراحل اولیه، قابل كنترل است. با توجه به نقش مهم اعتقادات و باورها در عملكرد زنان و اهمیت انجام رفتارهای غربال‌گری در تشخیص زودرس سرطان پستان، این مطالعه به منظور تعیین رفتارهای غربال‌گری سرطان پستان بر حسب مراحل آمادگی و ارتباط آن با عوامل روانی- شناختی در معلمین زن شهر اصفهان انجام شد.  روش‌ها‌ در این مطالعه توصیفی- تحلیلی از نوع مقطعی، 392 نفر از معلمین زن منطقه 3 شهر اصفهان به روش نمونه‌گیری تصادفی خوشه‌ای سهمی مورد بررسی قرار گرفتند. برای تعیین مراحل تغییر رفتارهای غربال‌گری سرطان پستان از ابزار مراحل تغییر Rakoweski و جهت بررسی عوامل روانی- شناختی از پرسش‌نامه مدل اعتقاد بهداشتی Champion گونه فارسی آن استفاده شد. داده‌ها پس از جمع‌آوری در نرم‌افزار SPSS نسخه 18 وارد و ضمن ارایه آمار توصیفی و تحلیلی در سطح معنی‌داری 05/0 = α تجزیه و تحلیل شد.  یافته‌ها میانگین سنی معلمین مورد مطالعه 1/6 ± 41/86 سال بود. توزیع افراد بر حسب مراحل تغییر خودآزمایی پستان به قرار زیر، 8/15 درصد پیش تفكر، 1/32 درصد تفكر، 6/17 درصد عمل، 8/27 درصد نگه‌داری و 6/6 درصد برگشت و برای رفتار غربال‌گری ماموگرافی به ترتیب 4/22 درصد، 5/31 درصد، 7/22 درصد، 6/17 درصد و 9/6 درصد در این مراحل بودند. آزمون ANOVA اختلاف معنی‌داری بین مراحل تغییر و منافع درك شده خودآزمایی پستان (02/0 = P)، انگیزه برای سلامتی (001/0 > P) و منافع درك شده ماموگرافی (001/0 > P) نشان داد. نتیجه‌گیری نتایج مطالعه نشان داد كه منافع درک شده، انگیزه برای سلامتی، بهترین پیش‌گویی کننده برای عملکرد خودآزمایی و ماموگرافی (مراحل عمل و نگه‌داری) هستند. از این رو توصیه می‌شود که با ارایه برنامه‌های آموزشی مناسب و بیان فواید و مزایای BSE و ماموگرافی در تشخیص زودرس سرطان پستان و ایجاد انگیزه مثبت برای سلامتی، زنان را در جهت مراحل عمل و نگه‌داری رفتار غربال‌گری سرطان پستان هدایت كنیم.

Title: بررسی غلظت نیتریت و نیترات در آب آشامیدنی مناطق تحت پوشش دانشگاه علوم پزشکی شهید بهشتی در استان تهران

http://hsr.mui.ac.ir/index.php/jhsr/article/view/393

مقدمه: امروزه افزایش غلظت ترکیبات نیتریت و نیترات در آب‌های طبیعی به خصوص منابع آب زیرزمینی شهرهای بزرگ، باعث ایجاد نگرانی‌هایی شده است. نیترات به عنوان یکی از شاخص‌های شیمیایی آلودگی آب از دیرباز مورد توجه بوده است. هدف از این مطالعه تعیین مقادیر نیتریت و نیترات در آب شرب استان تهران در مناطق تحت پوشش دانشگاه علوم پزشكی شهید بهشتی بود. روش‌ها: این مطالعه از نوع توصیفی- مقطعی با هدف تعیین مقادیر نیتریت و نیترات در آب آشامیدنی مناطق مربوطه در استان تهران صورت گرفت. بر اساس نتاج حاصل از مطالعات قبلی، در این بررسی به طور تصادفی تعداد 105 نقطه از مناطق تحت پوشش دانشگاه علوم پزشكی شهید بهشتی انتخاب و بعد از نمونه‌برداری، به روش اسپکتروفتومتری مورد آزمایش قرار گرفت. یافته‌ها: در آب آشامیدنی این مناطق، نیترات با میانگین mg/l 09/18 و انحراف‌ معیار mg/l 67/22 از حداکثر 5/87 تا حداقل mg/l 65/2 و مقدار نیتریت با میانگین µg/l 30/24 و انحراف معیار µg/l 72/52 از حداقل صفر تا µg/l 330 متغیر است. نتیجه‌گیری: گر چه میانگین غلظت نیترات در اكثر نمونه‌ها از 50 میلی‌گرم در لیتر کمتر است، كه استاندارد سازمان جهانی بهداشت و آخرین استاندارد ملی کشور می‌باشد، ولی با این وجود حفاظت از منابع آب زیرزمینی در برابر ورود آلایندها و همچنین انجام مطالعات فراگیر جهت پایش کیفیت شیمیایی منابع آبی و ارایه برنامه‌های عملی جهت تسریع در اجرای شبکه‌های جمع‌آوری و تصفیه فاضلاب، به منظور جلوگیری از بالاتر رفتن مقادیر این یون‌ها در آب، ضروری به نظر می‌رسد.

Title: عوامل مؤثر بر افت تحصیلی، از دیدگاه دانشجویان دانشگاه علوم پزشکی اصفهان

http://hsr.mui.ac.ir/index.php/jhsr/article/view/394

مقدمه: افت تحصیلی یکی از عمده‌ترین مشکلات مراکز آموزش عالی کشور به حساب می‌آید. این مطالعه با هدف بررسی عوامل مؤثر بر افت تحصیلی (عوامل خانوادگی، عوامل مرتبط با فراگیر، عوامل مرتبط با آموزش‌گر، عوامل اقتصادی- اجتماعی، محیط آموزشی، محتوای آموزشی)، از دیدگاه دانشجویان انجام شد. روش‌ها: این مطالعه از نوع توصیفی- تحلیلی است که در آن 280 نفر از دانشجویان دانشگاه علوم پزشکی اصفهان به صورت نمونه‌گیری خوشه‌ای چند مرحله‌ای انتخاب و مورد مطالعه قرار گرفتند. مشخصات دموگرافیک و دیدگاه دانشجویان در خصوص عوامل مؤثر بر افت تحصیلی به وسیله پرسش‌نامه‌ای که پیش از انجام مطالعه روایی و پایایی آن مورد تأیید قرار گرفته بود، جمع‌آوری گردید و در تجزیه و تحلیل داده‌ها از نرم‌افزار SPSS نسخه 15 استفاده شده است. یافته‌ها: مهم‌ترین عوامل مؤثر بر افت تحصیلی از دیدگاه دانشجویان به ترتیب محتوای آموزش (63/0 ± 23/4)، عوامل مرتبط با آموزش‌گر (55/0 ± 88/3)، محیط آموزش (62/0 ± 63/3)، عوامل خانوادگی (6/0 ± 53/3)، عوامل اقتصادی– اجتماعی (69/0 ± 45/3) بود. بین دیدگاه دانشجویان در دو جنس و حیطه آموزش‌گر (03/0 = P) و محیط اجتماعی و اقتصادی (003/0 = P) اختلاف معنی‌داری وجود داشت. علاوه بر آن نتایج اختلاف معنی‌داری بین دیدگاه دانشجویان با سن، وضعیت تأهل و وضعیت شغلی آنان نشان نمی‌دهد(05/0 < P). نتیجه‌گیری: توجه بیشتر به محتوای آموزشی، عوامل مرتبط با آموزش‌گر و همچنین محیط آموزشی، علاوه بر پیش‌گیری از افت تحصیلی دانشجویان از اتلاف وقت و هزینه‌های جاری مراکز آموزش عالی کشور جلوگیری و سبب بهبود و توسعه سیستم آموزش کشور می‌شود.

Title: بررسی تجزیه بیولوژیكی 2، 3، 7، 8 -تتراکلرو دی بنزو- P- دیوکسین در رآکتور بی‌هوازی پر و خالی شونده به طور متوالی (ASBR

http://hsr.mui.ac.ir/index.php/jhsr/article/view/396

مقدمه و هدف: 2، 3، 7، 8 -تترا کلرو دی بنزو- پی- دیوکسین (TCDD یا Tetra chlorine dibenzo-P-dioxin) به عنوان سمی‌ترین ترکیب شناخته شده است که به دلیل سمیت زیاد، پایداری و مقاومت بالایی که در محیط دارد، از اهمیت بالایی برخوردار می‌باشد. از این رو هدف از انجام این مطالعه بررسی تجزیه بیولوژیکی دیوکسین TCDD در رآکتور ASBR (Anaerobic sequencing batch reactor) است. روش بررسی: این مطالعه با استفاده از یک رآکتور بی‌هوازی پر و خالی شونده متوالی (ASBR) شیشه‌ای به حجم 3 لیتر انجام شد. سوبستره اصلی و کمکی به ترتیب TCDD و متانول بود. رژیم بهره‌برداری از رآکتور شامل چهار مرحله تغذیه، واکنش، ته‌نشینی و تخلیه بود. آزمایشات COD بر روی نمونه‌های سوبستره ورودی و پساب خروجی بر اساس روش‌های استاندارد، آنالیز TCDD بر روی نمونه‌های فوق به روش کروماتوگرافی گازی و آشکارساز ECD و اندازه‌گیری بیوگاز توسط دستگاه گازمتر تر انجام شد. یافته‌ها: بهتـرین عملکـرد رآکتور ASBR در حذف COD، TCDD و بیوگاز تولیدی به میزان 35/99 درصد، 5/97 درصد و 32/2 لیتر در روز بوده، که به ترتیب در بارگذاری‌های آلی gCOD/L.d 5/2 و TCDD برابر ng/L.d 100 اتفاق افتاد. شواهد نشان داد که در طی افزایش بارگذاری در مراحل اول مطالعه، مقدار افت pH در حدود 1 واحد بوده، اما پس از راه‌بری طولانی مدت، pH رآکتور به2/7 رسید که نشان‌گر ثبات عملکرد رآکتور بی‌هوازی است. نتیجه‌گیری: تجزیه بیولوژیکی TCDD در بارگذاری حداکثر حدود gCOD/L.d 5/2 با تزریق مقدار ng/l TCDD 200 در رآکتور مورد استفاده در این مطالعه امکان‌پذیر است.

Title: بررسی تأثیر کارگاه آموزشی بر آگاهی مربیان آموزشگاه‌های بهورزی استان اصفهان در زمینه فرایند یاددهی و یادگیری

http://hsr.mui.ac.ir/index.php/jhsr/article/view/398

مقدمه: آموزش بهورزان به دلیل مسؤولیت‌های آموزشی و خدماتی این گروه و همچنین برقراری ارتباط مستقیم با گیرندگان خدمات از اهمیت ویژه‌ای برخوردار است. به نظر می‌رسد که آموزش و توانمند‌سازی مربیان آموزشگاه‌های بهورزی در زمینه فرایند‌های یاددهی و یادگیری مانند شیوه‌‌های تعیین اهداف آموزشی، استفاده از تکنولوژی آموزشی مناسب و ... می‌تواند گامی مؤثر در جهت تربیت نیروهای کارامدتر و همچنین کاهش هزینه‌ها باشد. از این رو این پژوهش با هدف بررسی تأثیر کارگاه آموزشی بر آگاهی مربیان آموزشگاه‌های بهورزی استان اصفهان در زمینه فرایند یاددهی و یادگیری طراحی شد.   روش‌ها: این مطالعه نیمه تجربی و ازنوع قبل و بعد بود که در آن کل جمعیت (107 نفر) به روش سرشماری مورد مطالعه قرار گرفتند. مطالعه طی سال 1389 و در مرکز بهداشت استان اصفهان انجام شد. ابزار گردآوری داده‌ها یک پرسش‌نامه محقق ساخته بود که بر اساس اهداف پژوهشی تنظیم شد. پایایی پرسش‌نامه از طریق آزمون Cronbach’s alpha با ضریب 75 درصد در فاز پایلوت بر روی 20 نفر مورد تأیید قرار گرفت. پرسش‌نامه‌ها قبل و پس از برگزاری کارگاه توسط گروه مخاطب تکمیل شد. داده‌های جمع‌آوری شده در نرم‌افزار SPSS و با استفاده از آزمون‌های آماری t زوجی، Mann- Witney، 2χ و همبستگی Pearson تجزیه و تحلیل شد.   نتایج: از 107 فرد شرکت کننده در کارگاه تعداد 42 نفر (3/39 درصد) مرد و تعداد 65 نفر (7/60 درصد) زن بود. دامنه سنی افراد شرکت کننده بین 24 الی 54 سال با میانگین و انحراف معیار 3/6 ± 6/37 سال بود. نتایج مطالعه نشان داد که آگاهی افراد تحت پژوهش در حیطه‌های مختلف فرایند یاددهی و یادگیری پس از برگزاری کارگاه آموزشی افزایش معنی‌دار پیدا کرده است (001/0 > P).   نتیجه‌گیری: با توجه به این که نتایج این پژوهش و سایر مطالعات مشابه نشان دهنده تأثیر مثبت رویکردهای مشارکتی از جمله کارگاه‌های آموزشی در یادگیری بهتر مباحث مربوط به فرایند یاددهی و یادگیری می‌باشند، پیشنهاد می‌شود که از این نوع روش‌های مشارکتی مانند بحث گروهی، بارش افکار و ... در آموزش‌های آتی بیشتر استفاده شود.

Title: الگوی اپیدمیولوژیک خودکشی در استان ایلام در سال 1384

http://hsr.mui.ac.ir/index.php/jhsr/article/view/399

مقدمه: خودکشی یک مشکل مهم بهداشت عمومی در اغلب کشورها است. شواهد نشان می‌دهند که سالانه نزدیک به یک میلیون نفر در سراسر جهان در اثر خودکشی جان خود را از دست می‌دهند. طبق آمار رسمی، روزانه 13 مورد خودکشی نیز در ایران رخ می‌دهد. هدف از انجام این پژوهش تعیین الگوی اپیدمیولوژیک خودکشی در استان ایلام و مطالعه برخی از عوامل مرتبط بر آن بود. مواد و روش‌ها: این مطالعه مقطعی در سال 1384 به انجام رسید. بدین ترتیب که داده‌های مربوط به تمام موارد خودکشی موفق ثبت شده در پزشکی قانونی استان ایلام در سال مورد نظر (88 مورد) به وسیله چک لیست جمع‌آوری و با استفاده از نرم‌افزار SPSS و با بهره‌گیری از آزمون‌های t و 2χ مورد تجزیه تحلیل قرار گرفت. یافته‌ها: از نظر جنسیت 60 درصد از موارد مرد و از نظر قومیت 90 درصد کرد بودند. میانگین و انحراف معیار سنی افراد خودکشی کرده برابر با 17 ± 2/29 سال بود. به علاوه، 2/60 درصد از واحدهای پژوهش مجرد، 1/34 درصد متأهل، 4/3 درصد مطلقه و 3/2 درصد از آنان بیوه بوده‌اند. همچنین، روش‌های مورد استفاده برای خودکشی در 49 درصد از موارد خودسوزی، 18 درصد اسلحه گرم و 18 درصد حلق‌آویز بوده است. نتیجه‌گیری: با توجه به پایین بودن میانگین سنی افراد خودکشی کرده، بالاتر بودن فراوانی خودکشی در افراد مجرد و بالاتر بودن فراوانی استفاده از روش‌های خشن به ویژه خودسوزی، ضرورت برنامه‌ریزی جامع در جهت کاهش بروز موارد خودکشی در استان ایلام توصیه می‌شود.

Title: تحلیل برخی عوامل پیش‌بینی کننده کیفیت زندگی در بیماران مبتلا به دیابت نوع 2

http://hsr.mui.ac.ir/index.php/jhsr/article/view/400

مقدمه: با عنایت به ماهیت مزمن بیماری دیابت و تأثیر قابل ملاحظه آن بر كیفیت زندگی بیماران مبتلا، مطالعه‌ای با هدف بررسی عوامل پیش‌بینی کننده کیفیت زندگی در این بیماران به منظور برنامه‌ریزی مداخلات در حوزه ارتقای سلامت طراحی شد. روش‌ها: این بررسی، یك مطالعه توصیفی- تحلیلی است که بر روی 140 بیمار دیابتی نوع 2 تحت پوشش مرکز دیابت ام‌البنین شهر اصفهان انجام شد. ابزار جمع‌آوری اطلاعات، پرسش‌نامه چند وجهی شامل اطلاعات دموگرافیک و مرتبط با بیماری (12 گویه)، ابزار استاندارد اندازه‌گیری دیسترس دیابت (17 گویه)، ابزار استاندارد اندازه‌گیری خودکارامدی بیماران دیابتی (8 گویه) و ابزار استاندارد کیفیت زندگی اختصاصی بیماران دیابتی (15 گویه) بود. اطلاعات پس از جمع‌آوری، توسط نرم‌افزار SPSS نسخه 5/11 با استفاده از آزمون‌های 2χ، t مستقل، آنالیز واریانس یک طرفه، ضریب همبستگی Pearson و تحلیل رگرسیون چند متغیره مورد بررسی قرار گرفت. یافته‌ها: متغیرهای دیسترس دیابت (001/0 < P) و خودکارامدی (001/0 < P) با کیفیت زندگی بیماران دیابتی ارتباط آماری معنی‌دار داشت. میزان R2 (توان پیش‌گویی کنندگی) در این مطالعه 66/0 بود. مدل رگرسیون چند متغیره، متغیرهای اثرگذار بر خودمدیریتی تعدیل شده برای سایر متغیرها را دو متغیر دیسترس دیابت (01/0  = P، 277/0- = ß) و خودکارامدی (001/0 < P، 161/0 = ß) نشان داد. نتیجه‌گیری: بر اساس نتایج این مطالعه ضروری است که در برنامه‌ریزی مداخلات ارتقای سلامت در حوزه دیابت، توجه کافی به منظور اثربخشی و کارامدی بیشتر مداخلات انجام شده، به دو متغیر خودکارامدی و دیسترس دیابت مبذول شود.

Title: بررسی روایی و پایایی نسخه فارسی ابزار توانمندسازی بیماران دیابتی نوع 2

http://hsr.mui.ac.ir/index.php/jhsr/article/view/401

مقدمه: با عنایت به شیوع به نسبت بالای دیابت در ایران، در اختیار داشتن ابزاری معتبر و پایا در مطالعات به منظور ارزیابی توانمندسازی بیماران دیابتی نوع 2 در راستای طراحی مداخلات کارامد ضروری به نظر می‌رسد. این مطالعه به منظور تسهیل در سیاست‌گذاری در حوزه سلامت با توجه به تغییر رویکرد مواجهه با این بیماری از رویکرد پزشکی به نوع توانمندی، طراحی و اجرا گردید. روش‌ها: اعتبار و پایایی نسخه فارسی ابزار توانمندسازی در بیماران دیابتی نوع 2 (DES-LF) با یک مطالعه مقطعی انجام گردید. اعتبار محتوا، پایایی و روایی فرهنگی  نسخه ایرانی DES-LF از طریق بررسی کمی (میانگین و انحراف معیار، تحلیل عامل، همبستگی Spearman) و کیفی (مصاحبه، بحث گروهی متمرکز) بر روی 160 بیمار دیابتی نوع 2 شرکت کننده در مطالعه، مورد ارزیابی و تحلیل قرار گرفت. یافته‌ها: پایایی و روایی این مقیاس و سه خرده مقیاس آن، با عناوین "مدیریت جنبه‌های روانی دیابت" (94/0 = a)، "ارزیابی نارضایتی و آمادگی برای تغییر" (96/0 = a) و "تنظیم و دست‌یابی به اهداف دیابت" (96/0 = a) از طریق تحیل روان‌سنجی تأیید شدند. با انجام آزمون test-retest روی 50 بیمار با فاصله زمانی دو هفته، نتایج مطالعه همبستگی درونی مناسبی را نشان داد (001/0 < P). شاخص بالینی كنترل متابولیك (HbA1c) در بیماران دیابتی نوع 2 به نوعی تأیید کننده اندازه‌گیری مناسب توانمندسازی به واسطه این ابزار بود (001/0 < P). نتیجه‌گیری: یافته‌های این مطالعه، روایی و پایایی نسخه فارسی DES-LF را برای تعیین مسیر آموزش توانمندسازی افراد مبتلا به دیابت نوع 2 و انجام مداخلات روانی- اجتماعی در میان این بیماران را تأیید می‌نماید.

Title: بررسی ارتباط بین میزان ذخایر آهن و آهن دریافتی از غذا با پروفایل لیپیدی در زنان سنین باروری

http://hsr.mui.ac.ir/index.php/jhsr/article/view/402

مقدمه: بعضی از مطالعات نشان داده‌اند که افزایش میزان ذخایر آهن حتی در محدوده طبیعی نیز ممکن است که باعث مستعد شدن فرد برای ابتلا به بیماری‌های قلبی عروقی شود. اختلالات لیپیدی نیز از جمله عوامل خطر اثبات شده بیماری‌های قلبی عروقی می‌باشند. حال این سؤال مطرح می‌شود که آیا میزان ذخایر آهن با پروفایل لیپیدی ارتباط دارد یا خیر؟ از این رو هدف از این مطالعه بررسی ارتباط بین میزان ذخایر آهن و آهن دریافتی از غذا با پروفایل لیپیدی است. روش کار: این مطالعه به صورت مقطعی در 82 نفر از دانشجویان و کارمندان خانم سالم دانشگاه علوم پزشکی اصفهان که در سنین باروری می‌باشند و پس از اعلام فراخوان برای شرکت در این مطالعه اعلام آمادگی نموده‌اند، انجام شده است. غلظت فریتین سرم، اجزای فراسنج لیپیدی، قند و انسولین خون در کلیه افراد اندازه‌گیری شد. اطلاعات مرتبط با رژیم غذایی افراد با استفاده از پرسش‌نامه نیمه کمی بسامد خوراک جمع‌آوری شد. تجزیه و تحلیل داده‌ها از طریق نرم‌افزار SPSS نسخه 18 و با استفاده از آمار توصیفی و تحلیلی انجام شد. یافته‌ها: با استفاده از آزمون همبستگی Pearson بین سطح فریتین سرم با غلظت تری گلیسیرید (006/0 = P، 278/0 = r)، کلسترول تام ( 008/0 = P، 267/0 = r) و قند خون (006/0 = P، 275/0 = r) ارتباط مثبت و معنی‌دار وجود داشت، ولی ارتباط فریتین سرم با HDL-c،  LDL-cو انسولین سرم معنی‌دار نبود. پس از تعدیل عوامل مخدوش‌گر فقط ارتباط فریتین سرم با سطح قند خون معنی‌دار باقی ماند (016/0 = P). قبل و بعد از تعدیل عوامل مخدوش‌گر، بین میزان هموگلوبین و هماتوکریت خون با غلظت اجزای فراسنج لیپیدی، قند و انسولین خون ارتباط معنی‌داری وجود نداشت. قبل و بعد از تعدیل عوامل مخدوش‌گر، بین میزان کل آهن، آهن هم و غیر هم دریافتی از غذا با غلظت اجزای فراسنج لیپیدی، قند و انسولین خون ارتباط معنی‌داری وجود نداشت. نتیجه‌گیری: بر اساس یافته‌های این مطالعه سطح فریتین سرم با غلظت قند خون ناشتا ارتباط مستقیم و معنی‌دار داشت، که بر ارتباط میزان ذخایر آهن با قند خون حتی در افراد سالم تأکید می‌کند. اگر چه یافته‌های این مطالعه حاکی از عدم وجود ارتباط معنی‌دار بین میزان ذخایر آهن و آهن دریافتی از غذا با اجزای فراسنج لیپیدی و انسولین است، ولی انجام مطالعات اپیدمیولوژیک بزرگ‌تر، در مردان و سایر گروه‌های سنی به منظور تأیید یا رد یافته‌های مطالعه حاضر پیشنهاد می‌شود.

Title: بررسی میزان آسیب‌های شغلی و آگاهی از احتیاط‌های استاندارد در مورد بیماری ایدز و هپاتیت در استادان، دانشجویان و کارکنان دانشکده دندانپزشکی دانشگاه علوم پزشکی اصفهان

http://hsr.mui.ac.ir/index.php/jhsr/article/view/403

مقدمه: کارکنان دندانپزشکی از آنجا که همیشه در تماس با خون و ترشحات آلوده به خون بیماران می‌باشند جزء گروه‌های پرخطر محسوب می‌شوند. بنابراین آگاهی آنان از احتیاط‌های استاندارد برای پیشگیری از بیماری‌های عفونی مثل هپاتیت و ایدز، حایز اهمیت می‌باشد. هدف این مطالعه، برآورد میزان آسیب‌های شغلی در گروه دندانپزشکی و آگاهی از راه‌های پیشگیری می‌باشد. روش‌ها: در این مطالعه مقطعی، 156 شرکت کننده (از بین استادان، دانشجویان و کارکنان آموزشی) با روش نمونه‌گیری اتفاقی طبقه‌بندی شده انتخاب شدند. سپس داده‌های جمع‌آوری شده با پرسش‌نامه، با استفاده از نرم‌افزار 16SPSS تجزیه و تحلیل شدند. یافته‌ها: در مورد آگاهی گروه‌های مختلف قابل توجه است که فقط 10 درصد شرکت کنندگان، که از دانشجویان بودند، آگاهی متوسط داشتند. 31/30 درصد از آسیب‌ها مربوط به جراحت با سر سوزن، 8/30 درصد مربوط به پاشیدن مایعات بالقوه عفونی در داخل چشم، دهان و یا بینی، 31/30 درصد مربوط به تجهیزات تیز و 6/8 درصد مربوط به تماس پوست آسیب دیده با مایعات بالقوه عفونی بود. نتیجه‌گیری: یافته‌های این مطالعه نشان داد آگاهی از احتیاط‌های استاندارد در گروه هدف نامطلوب بود. بنابراین اجرای برنامه‌های آموزشی مستمر، بسیار ضروری به نظر می‌رسد.

Title: کنترل استرس گرمایی در سکوي ذوب‌ريزي يکي از کارخانجات ذوب فلزات تهران

http://hsr.mui.ac.ir/index.php/jhsr/article/view/404

مقدمه: استرس حرارتي يکي از آلاينده‌هاي محيط کار است که مي‌تواند مشکلات عديده‌اي را براي کارگران فراهم آورد. هدف از اين تحقيق، کنترل استرس حرارتي در سکوي ذوب‌ريزي يکي از کارخانجات ذوب فلزات تهران مي‌باشد. روش‌ها: در اين مطالعه تجربي، به منظور بهبود شرايط کار در سکوي ذوب‌ريزي يکي از کارخانجات ذوب فلزات، راهکارهايي ارایه گرديد. به منظور ارزيابي تدابير کنترلي، دو مرحله اندازه‌گيري، قبل و بعد از اجراي آن‌ها صورت گرفت و از شاخص WBGT براي ارزيابي شرايط محيطي استفاده شد. در اين تحقيق از دماسنج شيشه‌اي، دماسنج تر چرخان، دماسنج گويسان استاندارد و دماسنج کاتای نقره اندود با فاکتور کاتای 420 و رديف سرد شوندگي 55-52 به ترتيب براي اندازه‌گيري دماي خشک، دماي تر طبيعي، دماي تابشي و سرعت جريان هوا استفاده گرديد. يافته‌ها: در اين سکوي ذوب‌ريزي، بعد از انجام اقدامات کنترلي، دماي تابشي از 04/44 به 8/35 و شاخص WBGT از 88/28 به 57/26 تقليل يافت. نتيجه‌گيري: گرماي تابشي مواد مذاب، عامل اصلي استرس حرارتي در کارگران سکوي ذوب‌ريزي مي‌باشد که با استفاده از سپرهای بازتاب دهنده، مقدار زيادي از اين استرس کاهش يافت. واژه‌‌‌های کليدی: استرس حرارتي، شاخص WBGT، ذوب فلزات، گرماي تابشي، سپر حفاظتی منعکس کننده

Title: ارزیابی تأثیر سه نوع ماسک تنفسی تصفیه کننده هوا بر روی ضربان قلب در بار کاری مختلف

http://hsr.mui.ac.ir/index.php/jhsr/article/view/406

مقدمه: با وجود استفاده گسترده وسایل حفاظت تنفسی در مشاغل مختلف، هنوز تأثیر این وسایل بر روی پاسخ‌های فیزیولوژیکی افراد به خوبی شناخته نشده است. از این‌رو هدف از این مطالعه بررسی تأثیر وسایل حفاظت تنفسی تصفیه کننده هوا بر روی ضربان قلب بود. روش کار: این مطالعه تجربی، تحت شرایط کنترل شده دما و رطوبت (57/0 ± 8/19 = WBGT) در اتاقک شبیه‌سازی شرایط جوی انجام شد. ضربان قلب 11 نفر با میانگین (انحراف معیار) سن (34/2) 1/24 سال، قد (2/4) 172 سانتیمتر و شاخص توده بدن (1/1) 4/22 در حالت استراحت (ضربان استراحت) و در هنگام استفاده از سه نوع ماسک تنفسی سوپاپ‌دار (کلاس فیلتراسیون 2FFP مدل 1021JFY ساخت شرکت APASCIANI)، نیم‌صورت (دو فیلتره مدل Duetta 3P با کارتریج 3P ساخت شرکت SAPASCIANI) و تمام‌صورت (مدل BN/2002TR ساخت شرکت SPASCIANI) در سه سطح فعالیت جسمانی سبک (سرعت 34/1 متر با شیب 0 درصد)، متوسط (سرعت 34/1 متر با شیب 5 درصد) و سنگین (سرعت 34/1 متر با شیب 10 درصد) اندازه‌گیری و ثبت گردید. مدت زمان فعالیت سبک، متوسط و سنگین به ترتیب 30، 30 و 20 دقیقه بود و به فاصله هر 5 دقیقه ضربان قلب ثبت گردید. داده‌ها با استفاده از آزمون‌های آماری Repeated measurement ANOVA و t زوج و با کاربرد نرم‌افزار 16SPSS انجام شد.   نتایج: میانگین (انحراف معیار) ضربان قلب 11 نفر در حالت عدم استفاده از ماسک در فعالیت سبک (1/13) 5/93، متوسط (1/18) 7/109 و سنگین (8/25) 6/119 و هنگام استفاده از ماسک سوپاپ‌دار در فعالیت سبک (7/9) 8/102، متوسط (0/16) 7/116 و سنگین (2/23) 1/132 و هنگام استفاده از ماسک تمام‌صورت در فعالیت سبک (7/14) 3/109، متوسط (4/17) 0/125 و سنگین (1/23) 1/140 ضربان در دقیقه بود. میانگین ضربان قلب در حین استفاده از سه نوع ماسک تنفسی با حالت عدم کاربرد ماسک در سطوح مختلف فعالیت جسمانی معنی‌دار بود (001/0 > P). همچنین میانگین ضربان قلب در هنگام استفاده از ماسک سوپاپ‌دار و نیم‌صورت در مقایسه با حالت استفاده از ماسک تمام‌صورت در سطوح مختلف فعالیت جسمانی نیز اختلاف معنی‌داری را نشان داد. نتیجه‌گیری: استفاده از ماسک‌های تنفسی در مقایسه با حالت عدم استفاده از ماسک در هر سه سطح فعالیت جسمانی، باعث افزایش قابل توجهی در ضربان قلب می‌شود. همچنین میانگین ضربان قلب در هنگام استفاده از ماسک تمام‌صورت در مقایسه با ماسک سوپاپ‌دار و نیم‌صورت افزایش می‌یابد.

Title: بررسی آگاهی، نگرش و رضایت‌مندی روستاییان شهر اصفهان نسبت به برنامه پزشک خانواده*

http://hsr.mui.ac.ir/index.php/jhsr/article/view/407

مقدمه: برنامه پزشک خانواده در سال ١٣٨٤ به منظور دسترسی راحت و مناسب به خدمات سلامت در روستاها و شهرهای با جمعیت زیر ٢٠٠٠٠ نفر به اجرا در آمد. این مطالعه با هدف بررسی آگاهی، نگرش و رضایت‌مندی روستاییان شهرستان اصفهان نسبت به برنامه پزشک خانواده انجام شده است. روش‌ها: در این مطالعه مقطعی 1200 نفر از افراد تحت پوشش برنامه پزشک خانواده در مناطق روستايی شهر اصفهان مورد بررسی قرار گرفتند. ابزار جمع‌آوری داده‌ها پرسش‌نامه‌اي محقق ساخته بود. روایی و پایایی پرسش‌نامه از طریق سنجش روایی صوری، روایی محتوایی و پایایی درونی (78/0 = α) بررسی گردید. پس از جمع‌آوری داده‌ها، به منظور تجزیه و تحلیل آن‌ها از نرم افزار 15SPSS استفاده شد. یافته‌ها: میانگین نمره آگاهی و نگرش افراد مورد مطالعه به ترتیب 9/15 ± 1/33 و 9/10 ± 4/50 بود. 4/46 درصد افراد مورد مطالعه از برنامه پزشک خانواده رضایت داشتند. کارمندان مراکز بهداشتی، درمانی (7/78 درصد) مهم‌ترین منابع کسب اطلاعات افراد روستایی از برنامه پزشک خانواده بودند. نتیجه‌گیری: بر اساس نتایج مطالعه حاضر، میزان آگاهی و نگرش افراد مورد مطالعه در سطح متوسط می‌باشد. با توجه به نقش مهم کارکنان بهداشتی در انتقال اطلاعات، به نظر می‌رسد اجرای برنامه‌های آموزشی توسط کارکنان بهداشتی سبب افزایش آگاهی و نگرش افراد تحت پوشش و در نهایت اثربخشی برنامه پزشک خانواده گردد.

Title: بررسی تأثیر جنس لباس کار بر شاخص استرین فیزیولوژیکی (PSI) مردان در شرایط آب و هوایی گرم در اتاقک شرایط جوی

http://hsr.mui.ac.ir/index.php/jhsr/article/view/410

مقدمه: لباس کار می‌تواند تبادلات حرارتی میان انسان و محیط را محدود کند و سبب ایجاد تنش‌های حرارتی، اختلالات جسمانی و روانی و کاهش راندمان کار و بهره‌وری گردد. این مطالعه با هدف بررسی تأثیر جنس لباس کار بر شاخص استرین فیزیولوژیکی (PSI یا Physiological Strain Index) مردان در شرایط آب و هوایی گرم در اتاقک شرایط جوی انجام گرفت. روش‌ها: این پژوهش به صورت مداخله‌ای بر روی 18 دانشجوی پسر در 16 حالت ترکیبی از چهار نوع لباس کار معمولی (1، 2، 3، 4)، دو سطح فعالیت (سبک و متوسط) و دو نوع شرایط آب و هوایی (گرم و خشک، گرم و مرطوب) انجام شد. ضربان قلب و دمای عمقی افراد به ترتیب با استفاده از دستگاه‌های پایش ضربان قلب (Polar RS100) و پایش دمای عمقی بدن از طریق گوش (Questemp II) اندازه‌گیری و ثبت شد. تجزیه و تحلیل داده‌ها با استفاده از آزمون تکرار مشاهدات توسط نرم‌افزار SPSS انجام گرفت. یافته‌ها: نتایج نشان داد که در شرایط گرم و مرطوب، کمترین مقدار PSI در فعالیت سبک و متوسط به ترتیب مربوط به لباس نوع 100 درصد پنبه و 30 درصد پنبه- 70 درصد پلی استر بود. در شرایط گرم و خشک، کمترین مقدار PSI در هر دو فعالیت، مربوط به لباس نوع 30 درصد پنبه- 70 درصد پلی استر بود. میانگین شاخص PSI در شرایط گرم و مرطوب و هنگام انجام فعالیت متوسط، برای انواع لباس‌ها اختلاف معنی‌داری را نشان داد (044/0 = P). نتیجه‌گیری: یافته‌های تحقیق نشان داد که در راستای کاهش استرین گرمایی برای شرایط گرم و مرطوب در سطح فعالیت سبک، لباس 100 درصد پنبه و در فعالیت متوسط لباس 30 درصد پنبه- 70 درصد پلی استر و در شرایط گرم و خشک لباس 30 درصد پنبه- 70 درصد پلی استر مناسب‌تر است.

Title: بررسی تأثیر آموزش جنسی بر کاهش تعارض زوجین شهر اصفهان

http://hsr.mui.ac.ir/index.php/jhsr/article/view/411

مقدمه: تعارض، پدیده‌ای غیر قابل اجتناب میان همسران می‌باشد. زوجین به دلیل همکاری و تصمیمات مشترکی که می‌گیرند، درجات مختلفی از استقلال و همبستگی را نشان می‌دهند. درجات بالای استقلال و همبستگی، باعث فروپاشی خانواده می‌شود. بررسی ارتباط میان تأثیر آموزش جنسی بر کاهش نمره كل تعارض زناشویی و ابعاد آن شامل ارتباط با همسر، رابطه جنسی و نقش‌های مساوات طلبانه، از اهداف تحقیق حاضر بود. روش‌ها: پژوهش حاضر، به روش نیمه تجربی و از نوع پیش آزمون- پس آزمون با گروه کنترل بود. نمونه آماری تحقیق 30 زوج (60 نفر) از زوجینی بودند که به شیوه نمونه‌گیری تصادفی از فرهنگ‌سرای خانواده شهر اصفهان انتخاب و به دو گروه آزمایش و کنترل تقسیم شدند. ابزار گردآوری داده‌ها پرسش‌نامه استاندارد تعارض زناشویی بر اساس مقیاس لیکرت و مشخصات دموگرافیک بود. یافته‌ها: نتایج پژوهش با آزمون تحلیل کوواریانس، حاکی از تأثیرآموزش جنسی در کاهش تعارض زناشویی (05/0 > P، 9/416 = F)، خرده مقیاس‌های ارتباط با همسر (05/0 > P، 2/75 = F)، رابطه جنسی (05/0 > P، 54/3 = F) و نقش‌های مساوات طلبانه (05/0 > P، 60/321 = F) بود. نتیجه‌گیری: آموزش جنسی می‌تواند با افزایش ارتباطات مطلوب عاطفی، احساسی در زوجین باعث افزایش رضایت و کاهش تعارض میان آن‌ها شود.

Title: ارزیابی آلودگی میکروبی آب میوه‌های دست‌ساز (غیرپاستوریزه) هویج و طالبی در مراکز فروش آب میوه شهر گرگان

http://hsr.mui.ac.ir/index.php/jhsr/article/view/412

مقدمه: در ایران فروش آب میوه‌های غیر پاستوریزه که به روش دستی تهیه می‌شود، رایج است. از آنجايي كه در تهيه و توزيع این فرآورده‌ها موازین بهداشتی به طور کامل رعایت نمی‌گردد، احتمال بروز عفونت‌های غذایی افزایش می‌یابد. هدف این مطالعه، ارزیابی آلودگی میکروبی آب میوه‌های دست‌ساز بود. روش‌ها: این مطالعه توصیفی- مقطعی در نیمه اول سال 1388 انجام گرفت. تعداد 100 نمونه آب هویج و آب طالبی به صورت تصادفی از مراکز فروش شهر گرگان جمع‌آوری و با رعايت استاندارد، به آزمایشگاه مواد غذایی منتقل گردیدند و از نظر انتروباکتریاسه و اشرشیاکلی آزمون شدند. داده‌ها پس از جمع‌آوری با استفاده از نرم‌افزار آماری SPSS آنالیز گردید. یافته‌ها: میزان آلودگی نمونه‌های آب هویج به انتروباکتریاسه و اشرشیاکلی به ترتیب برابر 29 و 17 درصد و میزان آلودگی نمونه‌های آب طالبی به انتروباکتریاسه و اشرشیاکلی به ترتیب 12 و 7 درصد بود. از کل 100 نمونه 33 درصد نمونه قابلیت مصرف نداشتند که سهم نمونه‌های آب هویج وآب طالبی به ترتیب 42  و 24 درصد بود. نتيجه‌گيری: منبع اصلی آلودگی میکروبی مراکز تهیه و توزیع آب میوه، عدم رعایت مقررات گندزدایی میوه‌ها و عدم شست‌وشوی صحیح ظروف تولید و نگه‌داری آب میوه‌ها بود. آموزش کارگران این مراکز برای کاهش میزان آلودگی ضروری است.

Title: تعيين شيوع اختلالات اسکلتي- عضلاني و عوامل مؤثر بر آن در بين کارگران کارگاه‌هاي تهيه مبل خام به روش ارزيابي سريع کل بدن (REBA)

http://hsr.mui.ac.ir/index.php/jhsr/article/view/413

مقدمه: صنعت مبل بخشي از صنايع نيمه سنگين کشور را تشکيل مي‌دهد که اغلب نقش کارکنان در فرايندهاي توليد اجتناب ناپذير است. لذا ارزيابي و تشخيص عوامل خطر اختلالات اسکلتي- عضلاني، اقدامي مهم در فعاليت‌هاي کنترلي و پيش‌گيرانه از این گونه اختلالات ناشي از کار محسوب مي‌شود. عملكرد توليد در بيشتر فعاليت‌هاي مبل سازي با جا به جا کردن بار سنگين، بزرگ و سخت همراه است و شيوع اختلالات اسکلتي- عضلاني از جمله عوارض شغلي در اين صنعت مي‌باشد. بنابراین اين پژوهش، با هدف تعيين ميزان شيوع اختلالات اسکلتي- عضلاني و تعيين عوامل خطر ايجاد کننده آن به روش تحليل شغلي ارزيابي سريع بدن (Rapid entire body assessment یا REBA) در کارگران صنعت مبلمان سازي انجام گرفت. مواد و روش‌ها: اين مطالعه از نوع توصيفي- تحليلي و مقطعي بود. به منظور بررسي وضعيت‌هاي انجام کار افراد در حرفه مبل سازي، 197 نفر از کارگران اين حرفه به صورت تصادفي انتخاب شدند. وضعيت بدني آن‌ها با روش تحليل شغلي ارزيابي سريع بدن (REBA) و پرسش‌نامه نورديک مورد ارزيابي قرار گرفت. در نهايت داده‌ها با استفاده از نرم‌افزار 16SPSS و آزمون‌هاي آماري t-test Independent، 2χ و Fisher’s exact test تجزيه و تحليل شد. يافته‌ها: شيوع اختلالات اسکلتي- عضلاني در اين صنعت بالا بود. همچنين مشخص گرديد که سابقه کار و ساعات کار روزانه از فاکتورهاي بسيار تأثير گذار، با بيشترين ارتباط در بروز اختلالات اسکلتي- عضلاني در اندام‌هاي نه گانه بوده‌اند (05/0 > P). از سوي ديگر نتيجه تحليل ارزيابي سريع بدن افراد نمونه نشان داد که وضعیت کاري و نوع وظيفه از پارامترهاي بسيار مهم در بروز اختلالات اسکلتي- عضلاني اين افراد بود. بحث و نتيجه‌گيري: نتايج اين بررسي نشان داد که شيوع اختلالات اسکلتي- عضلاني در اندام‌هاي کمر، زانو، پا، دست و مچ دست بالا بوده است. همچنین وضعيت‌هاي بدن کارگران اين کارگاه‌ها نياز به اصلاح داشت. لذا پيشنهاد شد که شرايط و ايستگاه‌هاي کاري آن‌ها بهينه سازي شود و همچنين دوره‌هاي آموزشي کوتاه مدت و متناوب براي آن‌ها تشکیل گردد.

Title: عوامل مرتبط با رفتارهای پیش‌گیری کننده از بیماری سالک در خانواده‌های ساکن در مناطق آندمیک بیماری سالک در شهر یزد بر اساس مدل بزنف

http://hsr.mui.ac.ir/index.php/jhsr/article/view/414

مقدمه: مدل بزنف یکی از کامل‌ترین مدل‌های ترکیبی جهت مطالعه و شناسایی رفتار و ایجاد رفتارهای جدید در جامعه است. بنابراین این پژوهش، با هدف توصیف عوامل مرتبط با رفتارهای پیش‌گیری کننده از بیماری سالک بر اساس مدل بزنف، در مناطق آندمیک شهر یزد طراحی و اجرا شد. روش‌ها: در این مطالعه مقطعی120 خانواده ساکن در دو منطقه آندمیک بیماری سالک در شهر یزد، با استفاده از نمونه‌گیری چند مرحله‌ای، انتخاب شدند. جامعه مورد مطالعه پرسش‌نامه‌ای را که بر اساس مدل بزنف تنظیم و روایی و پایایی آن تأیید شده بود، طی مصاحبه تکمیل نمودند. تجزیه و تحلیل داده‌ها با آمار توصیفی و استنباطی انجام شد. یافته‌ها: آگاهی، نگرش و رفتار آزمودنی‌ها در سطح مطلوبی نبود. قصد استفاده از پشه‌بند آغشته به سم در سطح خیلی پایینی قرار داشت. آگاهی از محل خرید وسایل حفاظتی و دریافت آموزش، مهم‌ترین عوامل قادر کننده برای انجام رفتار بودند. تأثیرگذارترین هنجارهای انتزاعی، رابطین و کارمندان بهداشتی بودند. رفتارهای پیش‌گیری کننده با قصد و عوامل قادر کننده همبستگی مستقیم معنی‌دار داشتند (05/0 > P). بین نگرش با آگاهی و قصد نیز همبستگی مشاهده شد (05/0 > r، 05/0 > P). نتیجه‌گیری: با توجه به پایین بودن سطح رفتارهای پیش‌گیری کننده از بیماری سالک و اهمیت عوامل قادر کننده در راستای انجام این رفتارها، فراهم کردن عوامل قادر کننده به ویژه از طریق آموزش رابطین بهداشتی می‌تواند در پیش‌گیری از سالک مؤثر واقع شود.

Title: مقایسه اثر مصرف شیر سویا و شیر گاو، بر عوامل خطر بیماری‌های قلبی- عروقی و عملکرد کلیه در بیماران مبتلا به نوع II مبتلا به نفروپاتی

http://hsr.mui.ac.ir/index.php/jhsr/article/view/415

مقدمه: نفروپاتی دیابتی یکی از عوارض مهم بيماري دیابت محسوب مي‌شود كه پیشرفت بیماری با دريافت‌هاي غذايي افراد مرتبط مي‌باشد. هدف این مطالعه، بررسی اثرات مصرف شیر سویا در مقایسه با شیر گاو بر عوامل خطرساز قلبی- عروقی و عملکرد کلیه در بیماران مبتلا به دیابت نوع II مبتلا به نفروپاتی بود. روش‌ها: این پژوهش از نوع کارآزمایی بالینی متقاطع تصادفی و کنترل شده بود که بر روی 25 بیمار مبتلا به دیابت نوع II مبتلا به نفروپاتی انجام شد. شرکت کنندگان به صورت تصادفی یک رژیم غذایی حاوی شیر سویا یا رژیم غذایی حاوی شیر گاو را به مدت 4 هفته مصرف نمودند. یک دوره دو هفته‌ای آب‌گیری (Washout) در بین دو دوره مداخله برقرار شد. اندازه‌گیری‌های تن‌سنجی و آزمایشات بیوشیمیایی خون و ادرار مطابق پروتکل استاندارد صورت گرفت. یافته‌ها: فشارخون سیستول با مصرف رژیم غذایی حاوی شیر سویا در مقايسه با شير گاو به صورت معنی‌داری کاهش داشت و حتی پس از تعدیل عامل مخدوش‌گر (ميزان دريافت كربوهيدرات) معنی‌دار باقی ماند (درصد تغییرات به ترتيب در گروه شير سويا و شيرگاو: 5/4- درصد در مقابل 89/5 درصد، 05/0 > P بود). سطح تری‌گلیسرید به دنبال مصرف شیر سویا کاهش معنی‌داری را نشان داد (درصد تغییرات 22/15- درصد در مقابل 37/2 درصد بود، 05/0 > P). این تغییرات بعد از تعدیل عامل مخدوش‌گر معنی‌دار نبود. تغییرات سایر فاکتورهای قلبی- عروقی و شاخص‌های عملکرد کلیوی بین دو رژیم حاوی شیر سویا و شیر گاو تفاوت معنی‌داری نداشت. نتیجه‌گیری: مصرف شیر سویا می‌تواند اثرات سودمندی را بر فشارخون سیستول در بیماران مبتلا به دیابت نوع II مبتلا به نفروپاتی داشته باشد.

Title: تأثیر مداخلات اصلاح شیوه زندگی در ارتقای سلامت رانندگان تاکسی در اصفهان (برنامه قلب سالم اصفهان)

http://hsr.mui.ac.ir/index.php/jhsr/article/view/416

مقدمه: در پژوهش حاضر، به بررسی تأثیر مداخلات 6 ساله اصلاح شیوه زندگی روی رانندگان تاکسی در شهرستان اصفهان، با هدف ارتقای سلامت پرداخته شد. روش‌ها: برنامه قلب سالم اصفهان، یک مطالعه مداخلاتی جامعه‌نگر بود که با هدف اصلاح عوامل خطر بیماری‌های قلبی- عروقی و ارتقای شیوه زندگی، بین سال‌های 1380 تا 1386 به اجرا در آمد. پژوهش حاضر روی 288 نفر از رانندگان تاکسی انجام شد. 171 نفر از جامعه مورد مطالعه از میان 6175 نفر بررسی شده در مرحله اول برنامه قلب سالم اصفهان انتخاب شدند. 117 نفر دیگر نیز از میان 4719 نفر بررسی شده در مرحله سوم برنامه قلب سالم اصفهان انتخاب گردیدند. این افراد مورد مداخلات متعددی در خصوص پیش‌گیری از عوامل خطرساز بیما‌ری‌های قلبی- عروقی و اصلاح شیوه زندگی، طی 6 سال قرار گرفتند. کلیه افراد مورد مطالعه در مراحل اول و سوم از نظر عوامل دموگرافیک، اندازه‌گیری فاکتورهای فیزیکی شامل فشارخون و فاکتورهای بیوشیمیایی مورد بررسی قرار گرفتند. نمونه‌های خون جهت بررسی فاکتور‌های بیوشیمیایی بررسی شد. یافته‌ها: با وجود این که میانگین سنی رانندگان بین سال‌های 86-1380 تفاوت معنی‌داری نشان نداد، اندیکس تغذیه‌ای و میزان تحرک به طور معنی‌داری کاهش یافت (0001/0 > P). شیوع پرفشاری خون در مراحل اول و سوم به ترتیب 1/15 و 1/12 درصد بود که تغییر معنی‌داری نشان نداد. در حالی که شیوع هایپرتری‌گلیسیریدمی به طور معنی‌داری از 9/42 به 4/27 درصد کاهش یافت (007/0 > P). همچنین شیوع هایپرکلسترولمی به طور معنی‌داری از 8/21 به 3/10 درصد کاهش یافت (01/0 = P). تغییرات سایر عوامل خطر معنی‌دار نبود. نتیجه‌گیری: با توجه به اهمیت شغل رانندگان تاکسی و تأثیر مداخلات بر کاهش اغلب عوامل خطرساز قلبی- عروقی و به منظور گسترش و پایدار نمودن مداخلات پیشنهاد شد که سیاست‌گزاران بخش سلامت نسبت به گسترش و نهادینه کردن این مداخلات، جهت پیش‌گیری و کنترل بیماری‌های قلبی اقدام نمایند.

Title: بررسی میزان آلودگی صدا و اثرات آزاردهندگی آن در بیمارستان‌های شهر همدان

http://hsr.mui.ac.ir/index.php/jhsr/article/view/418

مقدمه: بیمارستان به عنوان محیط ارایه خدمات درمانی، تحت تأثیر منابع آلودگی صدا قرار دارد که می‌تواند از جنبه بهداشتی و آسایشی بر بیماران و کارکنان تأثیر منفی داشته باشد. علاوه بر نیاز کارکنان به محیطی آرام برای خدمت‌دهی مطلوب، توجه به آسایش بیماران در زمان بستری در روند بهبود بیماری آنان بسیار حایز اهمیت است. هدف این مطالعه بررسی میزان آلودگی صدا در بخش‌های مختلف بیمارستان و تعیین میزان آزار صوتی در بین کارکنان آن بود. روش‌ها: تراز معادل فشار صوت در طول روز در 302 ایستگاه تعیین شده در بخش‌های مختلف و محوطه 8 بیمارستان شهر همدان با استفاده از دستگاه صداسنج 1358-TES اندازه‌گیری گردید. همچنین پرسش‌نامه استاندارد شده‌ای با ضریب پایایی 991/0 جهت بررسی میزان شیوع آزار صوتی در بین 186 نفر از کارکنان توزیع و تکمیل گردید. يافته‌ها: میانگین تراز معادل فشار صوت در فضای داخل و خارج بیمارستان‌ها به ترتیب برابر با dB(A) 8/55 و dB(A) 2/56 بود و در داخل بیمارستان‌های آموزشی و غیر آموزشی، به ترتیب برابر با  dB(A)57 و  dB(A)54 بود. به صورت کلی 17 درصد کارکنان احساس آزار صوتی خفیف، 22 درصد آزار صوتی متوسط، 16 درصد آزار صوتی شدید و 45 درصد آزار صوتی بسیار شدید داشتند. علاوه بر این میزان آزار صوتی در زنان 10 درصد بیشتر از مردان تعیین گردید. نتیجه‌گیری: با توجه به حدود مجاز استاندارد صدای کشور در بیمارستان، وجود آلودگی صدا در بیمارستان‌های مورد مطالعه آشکار است. بر این اساس افزایش آگاهی کارکنان و مسؤولین بیمارستان‌ها و جامعه نسبت به اهمیت پایین نگاه داشتن سطح صدا، بهسازی محیط بیمارستان و یا تغییر مکان و نوسازی از پیشنهادهای این مطالعه بود.

Title: بررسی تأثیر مداخله آموزشی بر آگاهی، نگرش و رفتار دانش‌آموزان پایه اول دبيرستان‌های پسرانه شهر بوانات در خصوص فعالیت جسمانی

http://hsr.mui.ac.ir/index.php/jhsr/article/view/417

مقدمه: فعالیت جسمانی منظم روی ابعاد سلامت جسمی، روانی و اجتماعی فرد و جامعه اثر مثبت دارد. با توجه به شیوع بیماری‌های غیر واگیر مانند فشارخون اولیه، پوکی استخوان و بیماری‌های قلبی- عروقی که کم تحرکی از عوامل زمینه‌ساز آن‌ها است، مسؤولین بهداشتی در صدد تشویق افراد به انجام فعالیت جسمانی بیشتر می‌باشند. رشد عادات مفید بهداشتی در طی مراحل کودکی و شروع نوجوانی بسیار بااهمیت است؛ چرا که رفتارهایی که در نوجوانی شروع می‌شوند تمایل به ادامه در بزرگ‌سالی دارند. هدف از این مطالعه تعیین تأثیر مداخله آموزشی بر آگاهی، نگرش و رفتار دانش‌آموزان پایه اول دبيرستان‌های پسرانه شهر بوانات در خصوص فعالیت جسمانی در سال 1389 بود. روش‌ها: در این مطالعه تجربی از نوع آینده‌نگر، از دانش‌آموزان دو دبیرستان واجد شرایط شرکت در مطالعه استفاده شد. تمام دانش‌آموزان یک دبیرستان به صورت تصادفی (تعداد 42 نفر) به گروه آزمون و نیز تمام دانش‌آموزان دبیرستان دیگر (تعداد 43 نفر) به گروه شاهد طبقه‌بندی شدند. ابزار گردآوری داده‌ها پرسش‌نامه چهار قسمتی شامل اطلاعات دموگرافیک، سؤالات مربوط به آگاهی، سؤالات مربوط به نگرش و پرسش‌نامه عملکرد فعالیت جسمانی بود. سه قسمت نخست پرسش‌نامه محقق‌ساخته بود كه برای تعیین روایی پرسش‌نامه، از روایی صوری و روایی محتوا استفاده شد. جهت تعیین پایایی سؤالات مربوط به قسمت آگاهی از آزمون- بازآزمون و نیز برای تعیین پایایی سؤالات قسمت نگرش از همسانی درونی (Cronbach's alpha) استفاده گردید. برای سنجش عملکرد (قسمت چهارم پرسش‌نامه) نیز از پرسش‌نامه خود گزارشی Garcia استفاده شد که قبلاً روایی و پایایی آن مورد آزمون قرار گرفته بود. پس از اجرای پیش آزمون، مداخله آموزشی برای گروه آزمون انجام شد. دانش‌آموزان دو گروه آزمون و شاهد بلافاصله بعد از پایان آموزش و همچنین یک ماه پس از پایان آموزش پی‌گیری شدند. داده‌هاي جمع‌آوری شده در اين پژوهش با استفاده از نرم‌افزار 18SPSS و آزمون‌های آماری Independent t-test ، Paired t-test، 2χ، Mann-Whitney test و آزمون ANOVA با تکرار مشاهدات، مورد تجزیه و تحلیل قرار گرفتند. مشارکت افراد در این مطالعه آگاهانه و داوطلبانه بود. یافته‌ها: تفاوت معنی‌داری بین گروه‌های آزمون و شاهد در میانگین نمرات آگاهی (934/0 = P)، نگرش (155/0 = P) و رفتار فعالیت جسمانی (387/0 = P) قبل از مداخله وجود نداشت. همچنین یافته‌ها حاکی از وجود تفاوت معنی‌دار در میانگین نمره آگاهی در پی‌گیری‌های بلافاصله (001/0 > P) و یک ماه بعد از انجام مداخله (001/0 > P) و وجود تفاوت معنی‌دار در میانگین نمره نگرش، بلافاصله (001/0 > P) و یک ماه بعد از انجام مداخله (01/0 = P) در گروه آزمون نسبت به گروه شاهد بوده است. میانگین نمره رفتار فعالیت جسمانی یک ماه بعد از انجام مداخله در هر دو گروه آزمون و شاهد افزایش معنی‌داری داشت (به ترتیب 01/0 = P و 001/0 > P). اما اختلاف میانگین نمره رفتار فعالیت جسمانی، بین دو گروه معنی‌دار نگرديد (39/0 = P). نتيجه‌گيري: نتایج این مطالعه حاکی از اثربخشی مداخله آموزشی و در نتیجه افزایش آگاهی و نگرش بیشتر دانش‌آموزان گروه آزمون در مقایسه با دانش‌آموزان گروه شاهد، در خصوص فعالیت جسمانی و در نتیجه افزایش رفتار فعالیت جسمانی آنان می‌باشد. اگر چه میانگین نمره فعالیت جسمانی در گروه شاهد نیز افزایش معنی‌داری داشت، انجام مطالعاتي دیگر با در نظر گرفتن و كنترل بيشتر متغیرهای مخدوش‌گر احتمالی پیشنهاد می‌گردد.

Title: بررسی تأثیر فراورده‌های میکروبی محلول بر روی گرفتگی غشای فیبر توخالی شناور

http://hsr.mui.ac.ir/index.php/jhsr/article/view/419

مقدمه: تولیدات میکروبی محلول نقش مؤثری در گرفتگی بیولوژیکی غشایی در بیورآکتورهای غشایی دارد. هدف از انجام این مطالعه، بررسی نقش و میزان تولیدات میکروبی محلول در گرفتگی بیولوژیکی غشایی در بیورآکتورهای مورد استفاده در تصفیه فاضلاب شهری بود. روش‌ها: جهت تعیین مقدار هیدرات کربن و پروتئین، به ترتیب از روش Lowry و فنل سولفوریک اسید استفاده شد. همچنین آزمایشات Biochemical oxygen demand (BOD)، Chemical oxygen demand (COD)، Suspended solid (SS) و کدورت، بر اساس استاندارد اجرای آزمایشات آب و فاضلاب انجام شد. همچنین فشار داخل غشاء در زمان‌های مختلف بهره‌برداری ثبت گردید. یافته‌ها: غلظت هیدرات کربن و پروتئین استخراج شده از مایع آب لجن (سوپرناتانت) به ترتیب در محدوده mg/l 8–3 و mg/l 6/7–5/2 به دست آمد. محدوده جامدات معلق مایع مخلوط در داخل بیورآکتور در محدوده g/l 3–2 و کدورت و جامدات معلق پساب خروجی به ترتیب در حدود NTU 01/0 و mg/l 5 بود. نتیجه‌گیری: هیدرات کربن استخراج شده از سوپرناتانت نسبت به پروتئین استخراج شده، نقش برجسته‌تری در گرفتگی بیولوژیکی غشایی در بیورآکتورهای غشایی داشته است.

Title: تبیین مصرف سیگار در بین دانشجویان دانشگاه علوم پزشکی گلستان بر اساس مدل بزنف

http://hsr.mui.ac.ir/index.php/jhsr/article/view/408

مقدمه: سالانه حداقل 5/4 میلیون نفر در دنیا بر اثر استعمال سیگار و بیماری‌های ناشی از مصرف آن جان خود را از دست می‌دهند. الگوی بزنف یکی از الگوهای جامع مطالعه رفتار است. این مطالعه با هدف تبیین وضعیت مصرف سیگار بر اساس سازه‌های الگوی بزنف در بین دانشجویان سیگاری مقیم خوابگاه‌های دانشگاه علوم پزشکی صورت گرفت. روش‌ها: در این مطالعه توصیفی از نوع مقطعی، تعداد 78 دانشجوی سیگاری به روش گلوله برفی در بهار سال 1390 وارد مطالعه شدند. ابزار گرد‌آوری داده‌ها پرسش‌نامه محقق ساخته‌ای بود که بر اساس سازه‌های الگوی بزنف تهیه شده بود. داده‌ها از طریق آمار توصیفی به کمک آزمون‌های Independent t-test و ANOVA تحلیل شدند. یافته‌ها: هم اتاقی سیگاری (2/46 درصد) و دوستان سیگاری (41 درصد) از مهم‌ترین هنجارهای انتزاعی مصرف سیگار بودند. دسترسی آسان (1/73 درصد)، سهولت مصرف (9/67 درصد) و ارزانی قیمت (8/62 درصد) از مهم‌ترین عوامل قادرساز بودند. در سازه نگرش، 8/30 درصد نگرش منفی، 6/43 درصد نگرش خنثی و 6/25 درصد نگرش مثبت به مصرف سیگار داشتند. نتیجه‌گیری: هنجارهای انتزاعی و عوامل قادرساز از مهم‌ترین عوامل مؤثر در مصرف سیگار می‌باشند. بنابراین باید در برنامه‌های ترک سیگار و پیش‌گیری از آن به اهمیت این عوامل توجه ویژه‌ای مبذول شود.

Title: بررسی عوامل مؤثر بر تأخیر در ایمن‌سازی کودکان زیر هجده ماه خرم‌آباد

http://hsr.mui.ac.ir/index.php/jhsr/article/view/420

مقدمه: عدم ایمن‌سازی کامل و به موقع کودکان در مقابل بیماری‌های قابل پیش‌گیری با واکسن، ممکن است سبب مرگ آنان گردد و یا عواقب بسیار تأثر انگیزی داشته، سبب معلول شدن کودکان شود. با توجه به اهمیت ایمن‌سازی و لزوم شناسایی دلایل تأخیر ایمن‌سازی در منطقه، اين مطالعه به منظور تعيين علل مؤثر در تأخير ايمن‌سازي کودکان زیر هجده ماه خرم‌آباد در طي اواخر سال 1389 و اوایل سال 1390 طراحي و اجرا گرديد. روش‌ها: اين مطالعه به صورت توصيفي، تحليلي- مقطعي، از ابتدای دی‌ماه 1389 لغایت ابتدای خرداد ماه 1390 در خرم‌آباد انجام گردید. جامعه آماري مورد مطالعه شامل كليه كودكان زیر هجده ماه بودند. نمونه‌گیری به روش سرشماری انجام شد. گردآوری اطلاعات به روش مصاحبه حضوری سازمان‌یافته با پرسش‌نامه محقق ساخته بود که روایی و پایایی آن به تأیید رسید. داده‌ها با استفاده از نرم‌افزار آماری 16SPSS و آزمون‌های آماری 2χ، ANOVA، هبستگی Pearson و رگرسیون خطی مورد تجزیه و تحلیل قرار گرفت. یافته‌ها: 6/41 درصد (84 نفر) مادران نام هیچ یک از واکسن‌هایی که به کودک تزریق می‌شود را نمی‌دانستند و تنها 9/9 درصد آن‌ها نام 4 تا 5 واکسن تزریقی را ذکر کردند. نداشتن وقت و بیمار بودن کودک بیش‌ترین دلایل تأخیر در ایمن‌سازی اعلام شد و میانگین مدت تأخیر بر حسب دلایل مختلف تأخیر، تفاوت معنی‌داری داشت. همچنین در میانگین مدت تأخیر بین سطوح مختلف تحصیلاتی مادران اختلاف معنی‌داری وجود داشت (01/0 > P). رابطه معنی‌دار خطی بین مدت تأخیر با سن مادر و تعداد کودک خانواده به دست آمد. نتیجه‌گیری: با توجه به دلایل تأخیر و ارتباط‌های آماری به دست آمده، لزوم تلاش بیشتر برای ارایه آموزش در زمان ارایه مراقبت و کاستن از فرصت‌های از دست رفته ایمن‌سازی ضروری می‌باشد.

Title: مقایسه پیامدهای سلامتی مرتبط با نوبت‌کاری در دو نظام 12 ساعته اقماری و 8 ساعته غیراقماری در صنایع پتروشیمی ایران

http://hsr.mui.ac.ir/index.php/jhsr/article/view/421

مقدمه: دو نوع برنامه نوبت کاری متداول در صنایع پتروشیمی کشور، برنامه‌های 12 و 8 ساعته بوده است که هر یک می‌تواند پیامدهای سلامتی گوناگونی برای فرد نوبت‌کار در پی داشته باشد. مطالعه حاضر با هدف، مقایسه پیامدهای سلامتی ناشی از کار در نظام‌های 12 ساعته اقماری و 8 ساعته غیراقماری در کارکنان نوبت‌کار صنایع پتروشیمی ایران انجام شده است.   روش‌ها: این مطالعه مقطعی، در 21 شرکت پتروشیمی وابسته به شرکت ملی صنایع پتروشیمی انجام گرفت. جمعیت مورد مطالعه 1630 کارگر نوبت‌کار (1034 نوبت‌کار شاغل در نظام غیراقماری و 596 نوبت‌کار شاغل در نظام اقماری) بود. داده‌های این مطالعه به وسیله پرسش‌نامه‌ای متشکل از سؤالاتی در زمینه ويژگي‌هاي دموگرافيک، نظام نوبت‌کاري و مشکلات سلامتی جمع‌آوری شد. تجزیه و تحلیل داده‌ها با استفاده از نرم‌افزار 5/11SPSS انجام گردید و مقدار آلفای کمتر از 05/0 به عنوان سطح معنی‌داری در نظر گرفته شد.   یافته‌ها: میانگین سابقه کار در نظام نوبتی برای نوبت‌کاران اقماری و غیراقماری به ترتیب 65/3 ± 80/3 و 92/6 ± 24/8 سال به دست آمد. شیوع مشکلات سلامتی در بین نوبت‌کاران اقماری بیش از غیراقماری بوده است و این اختلاف در مورد اختلالات گوارشی و اسکلتی- عضلانی معنی‌دار به دست آمد.   نتیجه‌گیری: نتایج نشان داد که باوجود جوان‌تر بودن و سابقه شغلی کمتر نوبت‌کاران اقماری، کار در این نظام 12 ساعته پیامدهای سلامتی بیشتری نسبت به نظام 8 ساعته غیراقماری ایجاد نموده است.

Title: كيفيت زندگي زنان مبتلا به ديابت نوع دو: بررسی نقش خودکارامدی

http://hsr.mui.ac.ir/index.php/jhsr/article/view/423

مقدمه: دیابت شایع‌ترین بیماری متابولیک در انسان است و عوارض مزمن متعددی نظیر اختلالات بینایی، کلیوی، عروقی و عصبی دارد که همگی کیفیت زندگی بیمار را تحت تأثیر قرار می‌دهد. در اين راستا خودكارامدي به عنوان يك عامل بسيار مهم شناخته شده است كه تا چه اندازه بيمار مي‌تواند مسایل مربوط به كنترل بيماري خود را شخصاً انجام دهد. لذا هدف از انجام اين مطالعه، بررسي ارتباط بين خودكارامدي و كيفيت زندگي بیماران زن مبتلا به دیابت نوع دو بود. روش‌ها: این پژوهش یک مطالعه توصيفي- مقطعي بود که بر روي 187 نفر از بيماران زن مبتلا به دیابت مراجعه كننده به مرکز تحقیقات ديابت شهر همدان انجام گرفت. نمونه‌گيري به صورت تصادفی ساده از بین مراجعین صورت گرفت. به منظور گردآوری اطلاعات از پرسش‌نامه کیفیت زندگی خلاصه شده سازمان جهانی بهداشت و پرسش‌نامه خودکارامدی درک شده در خصوص کنترل بیماری استفاده شد. داده‌ها با استفاده از نرم‌افزار آماری 13SPSS مورد تجزیه و تحلیل قرار گرفت. یافته‌ها: ميانگين سال‌هاي ابتلا به ديابت در شرکت‌کنندگان 86/9 با انحراف معیار 77/6 سال بود. به طور كلي میانگین نمره كيفيت زندگي 92/79 (نمره کل 130) بود. بين شغل، سال‌های ابتلا به بیماری و میزان تحصیلات با كيفيت زندگي از نظر آماري رابطه معنی‌داري مشاهده شد (05/0 > P). همچنین بین خودکارامدی و کیفیت زندگی بیماران (05/0 > P) رابطه معنی‌داري دیده شد. نتیجه‌گیری: نتايج حاصل از مطالعه حاضر ارتباط معنی‌داري را بين خودكارامدي و كيفيت زندگي نشان داد. در این خصوص پرستاران و سایر پرسنل بهداشتی- درمانی می‌توانند با آموزش‌های مناسب خود نقش مهمي را در افزايش ميزان اطمينان در انجام رفتارهاي مراقبت از خود و به تبع آن بهبود كيفيت زندگي بیماران داشته باشند.

Title: بررسی غلظت الکیل بنزن سولفونات خطی در رودخانه زاینده‌رود و چاه‌های حاشیه آن در سال 1386

http://hsr.mui.ac.ir/index.php/jhsr/article/view/424

مقدمه: سورفاکتانت‌ها ترکیبات شیمیایی آلی هستند که به علت کاهش کشش سطحی آب موجب افزایش قدرت پاک‌کنندگی آب می‌شوند. LAS از دسته سورفاکتانت‌های آنیونی است که در منابع آب‌های سطحی می‌توانند مشکلات زیست محیطی، به خصوص پدیده اتریفیکاسیون را به وجود آورند. هدف این مطالعه تعيين غلظت LAS در رودخانه زاینده‌رود حد فاصل سد چم آسمان تا پل زیار، تعیین ارتباط بین LAS با ساير پارامترهای اندازه‌گیری شده (COD، BOD5، pH، EC و فسفر) و تأثیرپذیری چاه‌ها از آب رودخانه بود. روش کار: جهت اندازه‌گیری مقدار LAS و دیگر پارامترهای مورد نظر، نمونه‌ها از رودخانه زاینده‌رود، چاه‌های اطراف زاینده‌رود و تصفیه‌خانه جنوب اصفهان جمع‌آوری شدند. EC، دما و pH توسط دستگاه پورتابل YSI و LAS، COD و فسفر توسط دستگاه اسپکتروفتومتر و BOD5 به روش Winkler تعیین شد. یافته‌ها: در رودخانه غلظت LAS، COD، BOD5، EC و فسفر به ترتیب 114/0-025/0، 41-2/5، 3/17-4/2، 913-284 و 32/1-025/0 mg/l در چاه‌ها 056/0-009/0، 14-1، 4/3-7/0، 2230-408 و 517/0-019/0 mg/l بود. غلظت LAS در ورودی و خروجی تصفیه‌خانه فاضلاب جنوب به ترتیب عبارت از 52/5 و 076/0 mg/l بودند. همچنین رابطه همبستگی Pearson نشان داد که بین غلظت LAS، BOD5 و غلظت فسفر رابطه مستقیم و بین تمامی پارامترها و فاصله چاه‌ها از رودخانه رابطه عکس وجود داشت. نتایج: شهر اصفهان و تصفیه‌خانه فاضلاب جنوب این شهر بیشترین تأثیر را در افزایش غلظت LAS رودخانه زاینده‌رود داشت. کارایی حذف LAS در تصفیه‌خانه فاضلاب جنوب بالاتر از 98 درصد به دست آمد و غلظت پارامترهای اندازه‌گیری شده در فصل پاییز به طور معنی‌داری بیشتر از فصل تابستان بود.

Title: محاسبه بار حاصل از مهم‌ترین عوامل مرگ در ایجاد "سال‌های از دست رفته عمر، YLL" در استان بوشهر

http://hsr.mui.ac.ir/index.php/jhsr/article/view/425

مقدمه: علاوه بر میزان‌های اختصاصی مرگ به علل مختلف که نشانگر اهمیت عوامل مختلف مرگ می‌باشند، شاخص سال‌های از دست رفته عمر YLL به دلیل مرگ زودرس، توسط علل مختلف مرگ نیز از اهمیت خاصی برخوردار می‌باشد، زیرا نه تنها به تعداد مرگ، بلکه به سن متوفیان در هنگام مرگ نیز بستگی دارد و هر چه سن متوفیان در هنگام مرگ کمتر باشد، تعداد سال‌های از دست رفته عمر افزایش پیدا می‌کند. این بررسی به منظورمحاسبه و مقایسه سال‌های از دست رفته عمر برای مهم‌ترین عوامل مرگ در جامعه مورد بررسی می‌باشد. بررسی برای داده‌های مرگ مربوط به استان بوشهر برای سال 1386 به انجام رسیده است .   روش بررسی: سال‌های از دست رفته عمر برای سه عامل اولیه مرگ و‌ میر در استان بوشهر برای مدت یك سال به تفکیک زنان و مردان بر اساس تعداد مرگ سالانه برای هر عامل مرگ جداگانه محاسبه شده است. روش آماری به کار گرفته شده بدین صورت است که امید به زندگی در هر سن که مرگ اتفاق افتاده، تعداد سال‌های از دست رفته عمر محسوب می‌شود، ولی با این تفاوت که ارزش سال‌های مختلف عمر یکسان نمی‌باشد و در سنین بالاتر تعداد سال‌های از دست رفته عمر به تناسب وزن کمتری به خود می‌گیرند، که به اصطلاح به این کارDiscounting  یا تخفیف گفته می‌شود. تعداد مرگ‌ها، سن افراد در زمان مرگ و امید به زندگی در سنین مختلف داده‌های اولیه برای محاسبات بوده‌‌‌اند. داده‌های مرگ و میر توسط مركز بهداشت استان در اختیار مجریان طرح قرار گرفته است.   نتایج: عوامل مرگ حوادث ترافیكی، بیماری‌های قلبی عروقی و سرطان‌ها به ترتیب بیشترین سال‌های از دست رفته عمر را با 9596 سال، 8602 سال و 4037 سال به خود اختصاص داده‌اند. كه برای عوامل مرگ بیماری‌های قلبی و سرطان‌ها اختلاف در دو جنس كم، اما برای عامل مرگ حوادث ترافیكی سال‌های از دست رفته عمر مردان حدود چهار برابر زنان بود. میزان YLL در هر هزار نفر نیز در كل جمعیت برای عامل مرگ حوادث ترافیكی، بیماری‌های قلبی عروقی و سرطان‌ها به ترتیب 7/10 و 6/9 و 5/4 سال برای هر هزار نفر بوده است.   نتیجه‌گیری: با توجه به این كه مرگ از حوادث ترافیكی بیشترین سال‌های از دست رفته عمر را در مردان جامعه باعث می‌شوند، مهم‌ترین عامل كاهنده امید به زندگی در جمعیت مردان می‌باشد كه می‌توان دلیل آن را جوان بودن نسبی متوفیان از حوادث ترافیكی برشمرد. در زنان عامل مرگ بیماری‌های قلبی عروقی بیشترین سال‌های از دست رفته عمر را باعث شده، در مجموع دو جنس نیز حوادث ترافیکی بیشترین سال‌های از دست رفته عمر را نسبت به دیگر عوامل مرگ در جامعه به خود اختصاص داده است. این تحقیق نشان داد كه برای سه عامل مرگ مورد بررسی مرتبه اهمیت آن‌ها بر اساس شاخص سال‌های از دست رفته عمر، متفاوت از مرتبه اهمیت آن‌ها بر اساس میزان‌های مرگ سالانه از هر عامل می‌باشد.

Title: تصفیه رواناب‌های شهری با استفاده از ستون شنی پوشش داده شده با نانوذرات اکسید آهن همراه با میدان مغناطیسی و بدون میدان مغناطیسی

http://hsr.mui.ac.ir/index.php/jhsr/article/view/426

مقدمه: افزایش سطوح نفوذناپذیر، علاوه بر افزایش حجم و پیک جریان، سبب افزایش نوع و مقدار آلاینده‌ها در رواناب شهری می‌شود. رواناب آلوده اثرات نامطلوب بسیاری را بر محیط زیست و آدمی می‌گذارد. هدف اصلی این پژوهش، بررسی کارایی شن پوشیده شده با نانوذرات اکسید آهن، بدون میدان مغناطیسی و با میدان مغناطیسی و نیز مقایسه این دو روش در تصفیه رواناب‌های شهری است. مواد و روش‌ها: فلزات سنگین موجود در رواناب با دستگاه جذب اتمی، کدورت توسط کدورت‌سنج و pH با استفاده از pH متر و نیترات و فسفات با دستگاه DR5000 تعیین شد. جهت تعیین اندازه و مشخصات نانوذرات از SEM و برای تعیین درصد ترکیبات موجود در شن از دستگاه آنالیز EDAX استفاده شد. یافته‌ها: تصاویر SEM و آنالیز EDAX نشان داد که نانوذرات اکسید آهن به خوبی بر روی شن، پوشش داده شده است. همچنین، در ستون شن پوشیده شده با نانوذرات اکسید آهن بدون میدان مغناطیسی، درصد راندمان حذف برای کدورت (8/90)، سرب (3/73)، روی (8/75)، کادمیوم (6/85)، فسفات (5/67) و نیترات (بدون حذف) و در ستون شن پوشیده شده با نانوذرات اکسید آهن همراه با میدان مغناطیسی، درصد راندمان حذف برای کدورت (7/95)، سرب (5/89)، روی (9/79)، کادمیوم (5/91)، فسفات (6/75) و نیترات (بدون حذف) به دست آمد. آنالیز آماری داده‌ها نشان داد که اختلاف معنی‌داری بین حذف آلاینده‌های مورد مطالعه در دو ستون وجود دارد. بحث و نتیجه‌گیری: فیلترهای شنی پوشیده شده با نانوذرات اکسید آهن کارایی بالایی در بهبود کیفیت رواناب‌های شهری داشتند، ولی در صورتی که این فرایند همراه با میدان مغناطیسی استفاده شود، راندمان تصفیه باز هم بیشتر خواهد بود. ضمن آن که این روش‌ها در مقایسه با بسیاری از روش‌های موجود ساده‌تر، کم هزینه‌تر و مؤثرتر می‌باشند.

Title: کاربرد الگوی اعتقاد سلامتی در برنامه آموزشی و تأثیر آن بر آگاهی و نگرش زنان از روش‌های غربال‌گری سرطان پستان

http://hsr.mui.ac.ir/index.php/jhsr/article/view/431

مقدمه: سرطان پستان شایع‌ترین عامل مرگ و میر ناشی از سرطان در بین زنان در سراسر جهان است. این بیماری زنان ایرانی را حداقل 10 سال جوان‌تر از زنان دیگر، در کشورهای توسعه یافته گرفتار می‌‌کند. تشخیص زودرس از طریق غربال‌گری تنها روش در دسترس برای زنان است. الگوی اعتقاد سلامتی یکی از الگوهایی است که به ارزش‌ها و اعتقادات افراد در ارتباط با سلامتی تمرکز دارد، به طور گسترده برای تبیین رفتارهای غربال‌گری افراد مورد استفاده قرار می‌گیرد. روش ها: در این مطالعه مداخله‌ای تعداد 180 نفر از زنان مراجعه كننده به مراكز بهداشتی درمانی از طریق نمونه‌گیری تصادفی انتخاب شده، به دو گروه شاهد و مورد تقسیم شدند و با استفاده از پرسش‌نامه خود ساخته مورد مصاحبه قرار گرفتند. پرسش‌نامه حاوی اطلاعات دموگرافیك، آگاهی از بیماری سرطان پستان، روش‌های غربال‌گری سرطان پستان (خودآزمایی سرطان پستان، معاینات بالینی توسط پزشک و ماموگرافی) و نگرش كه بر اساس چارچوب الگوی اعتقاد سلامتی (حساسیت، شدت، موانع و منافع درک شده تنظیم شده بود. در مرحله اول مداخله آموزشی پس از جمع‌آوری داده‌های اولیه انجام شد که در مرحله دوم مجدداً یک ماه پس از مداخله اطلاعات از هر دو گروه جمع‌آوری شد. داده‌ها با استفاده از آزمون t تست مورد تحلیل قرار گرفت. یافته‌ها: میانگین سن زنان در این مطالعه در گروه مورد و شاهد به ترتیب (96/11) 45/35 و (47/10) 45/36 بود. بر اساس نتایج به دست آمده، میانگین آگاهی و تمام اجزای الگو اعتقاد به سلامتی بعد از مداخله افزایش معنی‌دار داشته است (001/0 < P). موانع درک شده از بین این اجزاء افزایش میانگین بالاتری را داشته است. نتیجه‌گیری: این مطالعه نیاز به ارایه برنامه‌های آموزشی آگاهی بخش از سرطان پستان در زنان را ضروری بخش می‌داند. نتایج این مطالعه بر برنامه‌های آموزشی و ارتقاء دهنده سیاست‌های سلامت عمومی زنان را مورد تأکید قرار می‌دهد.

Title: مقایسه روش تخمیر چند لوله‌ای متداول با روش A-1در ارزیابی كیفیت میکروبی لجن دفعی تصفیه خانه‌های فاضلاب

http://hsr.mui.ac.ir/index.php/jhsr/article/view/433

مقدمه: با وجود مزایای لجن دفعی تصفیه‌خانه‌های فاضلاب جهت کاربرد در زمین به عنوان حالت دهنده و حاصلخیز کننده خاک، نگرانی‌های زیادی در ارتباط با خطرات مرتبط با لجن وجود دارد. لجن حاوی طیف وسیعی از ارگانیسم‌های بیماری‌زا و مواد شیمیایی مضر مانند فلزات سنگین است، که می‌تواند برای سلامتی انسان مخاطره‌آمیز باشد. بنابراین پایش میكروبی لجن وظیفه‌ای مهم برای سازمان‌های مسؤول جهت ارزیابی كارایی واحدهای تصفیه و همچنین قابلیت کاربرد آن در زمین می‌باشد. روش‌ها: در این مطالعه کیفیت میکروبی لجن دفعی دو تصفیه‌خانه فاضلاب شمال و جنوب اصفهان با روش متداول تخمیر چند لوله‌ای (MTF یا Multiple tube fermentation technique) و روش محیط کشت A-1 مورد ارزیابی و مقایسه قرار گرفت. در این پژوهش 30 نمونه به روش استاندارد از لجن دفعی برداشت شد. بعد از اندازه‌گیری دمای نمونه و دمای محیط نمونه‌ها به آزمایشگاه منتقل شد. سپس نمونه‌ها از نظر دانسیته کلیفرم‌های مدفوعی از طریق دو روش ذکر شده آزمایش شدند. جامدات کل (TS یا Total solids) و جامدات فرار (VS یا Volatile solids) نمونه‌ها نیز اندازه‌گیری شد. یافته‌ها: نتایج کشت میکروبی نمونه‌ها به تفکیک تصفیه‌خانه‌های مورد بررسی نشان داد که در تصفیه‌خانه شمال و جنوب مقادیر کلیفرم مدفوعی تمام نمونه‌ها بالاتر از استاندارد کلاس A و B می‌باشد. میانگین شمارش شده كلیفرم‌های مدفوعی به روش MTF و A-1 به ترتیب برابر با 14 + E 25/3 و 14 + E 1/2 MPN/g بود. نتایج آنالیز آماری، این اختلاف را معنی‌دار نشان داد. بحث و نتیجه‌گیری: با توجه به نتایج این تحقیق، روش  A-1می‌تواند جانشینی مناسب برای روش متداول MTF جهت پایش كیفیت لجن دفعی تصفیه‌خانه‌های فاضلاب باشد. این روش با مزایایی نظیر سادگی و سرعت در ردیابی كلیفرم‌های مدفوعی در عرض 24 ساعت می‌تواند اطلاعات مفیدی را در ارتباط با دانسیته كلیفرم‌های مدفوعی در لجن فراهم نماید.

Title: تأثیر آموزش تغذیه و بهداشت مواد غذایی بر آگاهی معلمین زن ابتدایی شهر فردوس

http://hsr.mui.ac.ir/index.php/jhsr/article/view/434

مقدمه معلمین یکی از گروه‌های مؤثر بر ارتقای سطح سلامت جامعه می‌باشند و ارایه آموزش‌های تغذیه توسط آنان به دانش‌آموزان علاوه بر تأثیر بر آگاهی دانش‌آموزان، می‌تواند باعث انتقال آموزش تغذیه به سطح خانوار باشد. این تحقیق با هدف تأثیر آموزش به روش مداخله‌ای بر آگاهی معلمین زن مدارس ابتدایی در زمینه تغذیه و بهداشت مواد غذایی انجام گرفته است. روش کار در این مطالعه نیمه تجربی تعداد 57 نفر از معلمین ابتدایی (زن) شهر فردوس انتخاب شدند. قبل از شروع آموزش، پرسش‌نامه در زمینه آگاهی در خصوص تغذیه و بهداشت مواد غذایی تکمیل و سپس کارگاه آموزشی 2 روزه برگزار شد و پس از 2 ماه مجدداً آزمون ثانویه از معلمین انجام شد. نتایج با استفاده از آزمون توصیفی و تحلیلی و توزیع زمان نمره آگاهی به وسیله آزمون t-test زوج شده، ANOVA و McNemar مورد تجزیه و تحلیل قرار گرفت. نتایج میانگین نمره آگاهی قبل از مداخله 98/10 درصد بود که بعد از مداخله به 2/18درصد افزایش یافت (0/001 < P). در مورد اهمیت مصرف صبحانه، آگاهی معلمین از 9/57 درصد در قبل از مداخله به 2/98 درصد بعد از مداخله افزایش یافت. نتیجه‌گیری استفاده از روش‌های مداخله‌ای آموزشی در مراکز تربیت معلم با همکاری مراکز بهداشت و آموزش و پرورش در خصوص تغذیه و بهداشت مواد غذایی می‌تواند عامل مؤثری در ارتقای سطح سلامت دانش‌آموزان باشد.

Title: بررسی سبک زندگی سالم معلمان شاغل در مدارس دولتی دخترانه ناحیه 4 آموزش و پرورش شهرستان اصفهان در سال 1389

http://hsr.mui.ac.ir/index.php/jhsr/article/view/436

مقدمه: نقش رفتارهای سالم مانند فعالیت بدنی، تغذیه و مدیریت استرس بر روی کاهش مرگ و میر و ابتلا بیماری‌ها به خوبی شناخته شده است. بنابراین مطالعه حاضر با هدف تعیین سبک زندگی سالم معلمان شاغل در ناحیه 4 آموزش و پرورش شهر اصفهان در سال 1389 انجام گرفت. روش‌ها: مشارکت کنندگان در این مطالعه مقطعی 96 معلم شاغل در ناحیه 4 بودند که به صورت تصادفی انتخاب شدند. ابزار جمع‌آوری داده‌ها پرسش‌نامه‌ای مشتمل بر دو بخش اطلاعات دموگرافیک و سبک زندگی سالم بود. در نهایت نیز داده‌ها با نرم‌افزار SPSS نسخه 18 مورد تجزیه و تحلیل قرار گرفت. یافته‌ها: میانگین سنی افراد 06/5 ± 26/40، میانگین شاخص توده بدنی 20/3 ± 08/25 بود. 8/96 درصد از افراد متأهل و 1/3 درصد مجرد بودند. 4/85 درصد از معلمان سبک زندگی مطلوب، 5/13 درصد نیمه مطلوب و 1 درصد نامطلوب داشتند. در ارتباط با تغذیه 74 درصد سبک زندگی مطلوب، 24 درصد نیمه مطلوب و 1/2 درصد نامطلوب داشتند. در ارتباط با فعالیت بدنی 76 درصد، سیگار کشیدن یا در معرض دود آن بودن 2/29 درصد و مقابله با استرس 9/21 درصد سبک زندگی نیمه مطلوب داشتند. نتیجه‌گیری: با توجه به نتایج برنامه‌ریزی‌ برای معلمان شاغل در مدارس، همچنین کسب اطلاعات در زمینه سبک زندگی سالم می‌تواند بسیار موثر واقع شود.

Title: بررسی عملکرد دانشجویان دانشگاه علوم پزشکی اصفهان در زمینه مصرف قرص‌های اکستازی بر اساس مدل اعتقاد بهداشتی در سال 89

http://hsr.mui.ac.ir/index.php/jhsr/article/view/435

مقدمه: مصرف قرص‌های اکستازی در سطح جهان 70 درصد افزایش داشته است و در حال حاضر مصرف آن از هروئین و کوکائین بیش‌تر است. بررسی‌های انجام شده حاکی از آن است که دانشجویان طبقه‌ای هستند که بیش از دیگر گروه‌ها از قرص‌های اکستازی استفاده می‌کنند و مصرف اکستازی در سال‌های اخیر در میان دانشجویان دانشگاه رشد صعودی داشته است. بنابراین، این مطالعه با هدف تعیین عملکرد دانشجویان دانشگاه علوم پزشکی اصفهان در زمینه مصرف قرص‌های اکستازی بر اساس مدل اعتقاد بهداشتی در سال 89 انجام گرفت.   روش ها: این پژوهش یک مطالعه توصیفی- تحلیلی است، که بر روی 267 نفر از دانشجویان دانشگاه علوم پزشکی اصفهان انجام گرفته است. ابزار گردآوری پرسش‌نامه‌ای روا و پایا مشتمل بر 3 قسمت (اطلاعات زمینه‌ای، اجزای مدل اعتقاد بهداشتی و عملکرد) بوده است، که با رعایت نکات اخلاقی تکمیل شده است. داده‌ها پس از جمع‌آوری با استفاده از نرم‌افزارSPSS  نسخه 18 و آزمون‌های آماری 2χ، t تست و kruskal-walis مورد تجزیه و تحلیل قرار گرفت.   یافته ها: میانگین سنی دانشجویان 61/1 ± 95/20 بود و (3/39) 105 مرد و (6/90) 242 مجرد بوده‌اند. (1/22) 59 نمونه‌ها قرص‌های اکستازی را دیده‌اند و (2/2) 6 افراد تاکنون از این قرص‌ها استفاده کرده‌اند. میانگین اجزای مدل نیز به ترتیب شامل حساسیت درک شده 68/19 ± 75/72، شدت درک شده 96/16 ± 58/84، منافع درک شده 49/23 ± 43/80، موانع درک شده 53/18 ± 65/81 بود. نتایج نشان داد که بین مصرف اکستازی با شدت درک شده و منافع درک شده رابطه معنی‌داری وجود داشت (001/0 ≤ P)، اما بین مصرف اکستازی با حساسیت درک شده و موانع درک شده رابطه معنی‌داری وجود نداشت (076/0 = P، 554/0 = P). در مورد راهنمای به عمل نیز دانشجویان رادیو و تلویزیون را مهم‌ترین راهنما می‌دانستند. نتیجه‌گیری: با توجه به نتایج حاصله جهت پیش‌گیری از مصرف قرص‌های اکستازی در دانشجویان علوم پزشکی با استفاده از مدل اعتقاد بهداشتی، باید برای بالا بردن حساسیت درک شده تلاش کرد و همچنین از نقش رادیو و تلویزیون نیز به عنوان مهم‌ترین راهنمای به عمل نباید غافل شد.

Title: مطالعه قابلیت فعالیت مجدد نوری باکتری‌های شاخص در پساب پرتوتابی شده تصفیه‌خانه فاضلاب شهری

http://hsr.mui.ac.ir/index.php/jhsr/article/view/438

مقدمه: فعالیت مجدد نوری میکروارگانیسم‌ها پس از گندزدایی با اشعه UV از معایب فن‌آوری فرابنفش در گندزدایی پساب است که در برخی موارد به چندین لگاریتم می‌رسد. هدف از انجام این مطالعه، ارزیابی امکان فعالیت مجدد نوری باکتری‌های شاخص (کلیفرم‌ها و استرپتوکک‌ها) به دنبال پرتوتابی پساب بود. روش‌ها: سه پایلوت با گزینه‌های مختلف تصفیه و گندزدایی شامل پساب ثانویه + لامپ فرابنفش کم فشار (LP) + لامپ فرابنفش فشار متوسط (MP)، زلال‌سازي + LP + MP و فيلتراسيون + LP + MP، طراحی و در محل تصفیه‌خانه فاضلاب شمال اصفهان نصب شد. پارامترهای میکروبی کلیفرم کل، کلیفرم مدفوعی و استرپتوکوک مدفوعی پس از پرتودهی با UV و پس از تابش نور مرئی و همچنین پارامتر TSS (Total suspended solids) وUVT  (UV-transmittance) در قبل و بعد از واحدها مورد آزمایش قرار گرفت. يافته‌ها: متوسط عبور اشعه در طول موج nm 254 از پساب ثانويه، زلال شده و فيلتر شده به ترتیب3/5، 34 و 51 درصد بوده است. بيش‌ترين فعاليت مجدد نوري كليفرم‌ها پس از گندزدايي پساب ثانويه با لامپ كم فشار (15 درصد) و كم‌ترين فعاليت مجدد نوري در گندزدايي پساب فيلتر شده با لامپ فشار متوسط (03/0 درصد) مشاهده شده است. نتيجه‌گيری: بر اساس نتایج حاصل از این مطالعه می‌توان دریافت که انتخاب نوع لامپ UV برای گندزدایی پساب تصفیه‌خانه فاضلاب با توجه به فعالیت مجدد نوری باکتری‌ها، فاکتور بحرانی است. ولی در دزهای کاربردی بیشتر از 2mWs/cm 230 برای باکتری‌های معلق پراکنده چندان مؤثر نیست.

Title: طراحي سامانه تجزيه و تحليل خطر و نقاط كنترل بحراني در سيستم تأمين و توزيع آب آشاميدني اصفهان از منبع تا نقطه مصرف

http://hsr.mui.ac.ir/index.php/jhsr/article/view/439

مقدمه: تأمين آب سالم و بهداشتي از نيازهاي اساسي افراد هر اجتماعي محسوب مي‌گردد که تنها با مديريت پيش‌گيرانه و جامع در برنامه ايمني آب آشاميدني مانند سيستم HACCP (Hazard analysis and critical control points) امكان‌پذير خواهد بود. HACCP يك سيستم كنترل فرايند پذيرفته شده بين‌المللي است كه شامل تشخيص و تعيين خصوصيات عوامل خطرساز بر روي محصول مورد فرايند و پيش‌بيني و اجرای اقدامات كنترلي جهت نگهداري و حفظ كيفيت مطلوب محصول در طول فرايند توليد از منبع تا نقطه مصرف است. هدف از انجام اين تحقيق، طراحي يك الگو و چارچوب جامع مديريت خطر بر اساس روش HACCP، به عنوان روشي براي مديريت كيفيت آب آشاميدني شهر اصفهان بود. روش‌ها: طي اين طرح مهم‌ترين خطرات و رويدادهاي خطرناك تأثيرگذار بر كيفيت آب در هر سه مقطع آب خام (رودخانه زاينده‌رود)، تصفيه‌خانه بابا شيخ‌علي و سيستم توزيع آب آشاميدني شهر اصفهان مورد پيش‌بيني، شناسایي و تجزيه و تحليل قرار گرفت و در قالب هفت اصل سامانه HACCP راهكارها و اقدامات عملي جهت پايش، كنترل و محدودسازي اين خطرات در مراحل مختلف ارایه شده است. اطلاعات مورد نياز از طريق بازديدهاي ميداني صورت گرفته در محدوده مورد مطالعه و نيز از طريق سازمان‌هاي مرتبط به دست آمد. يافته‌ها: پتانسيل ايجاد خطرات مختلف، حاصل از تغييرات مقطعي كيفيت آب مورد تصفيه در اثر تغييرات فصلي، تخليه آلاينده‌هاي مختلف به خصوص توسط جوامع روستایي و مراكز تفريحي حاشيه زاينده‌رود، حوادث طبيعي و غير مترقبه، حوادث عمدي و خراب‌كارانه، عدم راهبري صحيح فرايندهاي فعلي و نيز عدم متناسب‌سازي فرايندهاي تصفيه و در نهایت وسعت و قدمت بالاي شبكه توزيع آب آشاميدني اصفهان بود. نتيجه‌گيري: جهت دستيابي به آب آشاميدني مطلوب و با كيفيت بالا، لزوم كنترل تخليه آلاينده‌ها در بالادست آبگير با توجه به روند رو به افزايش ميزان آلودگي آب زاينده‌رود، متناسب‌سازي فرايندهاي تصفيه موجود و حفظ كيفيت آب آشاميدني در سيستم توزيع، ضروري مي‌باشد.

Title: تغییرات رطوبت، چربی و شاخص‌های اکسایش چربی گوشت در حین تهیه کباب کوبیده ایرانی

http://hsr.mui.ac.ir/index.php/jhsr/article/view/441

مقدمه: کباب کوبیده غذاي رايج و سنتي ايراني است. فرایند حرارتی در طول تهيه كباب موجب خروج رطوبت و بخشی از چربی از گوشت می‌شود. نسبت رطوبت و چربی جدا شده از گوشت، تعیین کننده میزان چربی در محصول نهايي است. بر اساس مطالعات اخير حرارت شديد، حضور اکسیژن و آهن در گوشت شرایط مناسبی را برای اکسایش چربی گوشت در طول تهيه گوشت‌هاي كبابي فراهم مي‌كند. هدف از انجام این مطالعه بررسی روند تغییرات رطوبت، ماده چرب، عدد پراکسید و عدد اسیدی در چربی گوشت در حین فرآوری به شکل کباب کوبیده ایرانی بود. روش‌ها: نمونه‌هاي گوشت از رستوران سلف سرويس دانشگاه علوم پزشكي اصفهان، در 5 نوبت و با فاصله زماني دو دقيقه در طول طبخ كباب تهيه شد. نمونه‌گيري سه مرتبه و با فاصله حداقل یک هفته تکرار گرديد. رطوبت به روش وزني و چربي با روش سوکسله اندازه‌گيري شد. جهت استخراج چربي از دو حلال پترولیم اتر و دی‌اتیل‌اتر استفاده شد. پس از تبخير حلال، عدد پراکسید چربي استخراج شده با روش يدومتري و عدد اسیدی با استفاده از تیتراسیون روغن با پتاس الکی اندازه‌گيري شد. یافته‌ها: در طول فرايند، رطوبت گوشت خام از16/1 ± 5/62 درصد تا 27/2 ± 5/45 کاهش پيدا کرد. بيش‌ترين نرخ كاهش رطوبت بين دقيقه دوم تا چهارم پخت مشاهده شد، ولي پس از دقيقه چهارم کاهش رطوبت با سرعت يكنواختي تا آخرين مرحله پخت ادامه پيدا كرد. در مقابل، چربی از 07/1 ± 5/7 درصد به 32/0 ± 7/15 با استفاده از حلال پتروليم بنزن و از 32/10 درصد تا 06/0 ± 44/19 درصد با استفاده از حلال دی‌اتیل‌اتر افزایش پیدا کرد. ميزان پراكسيد و عدد اسيدي محاسبه شده در روغن استخراجي از گوشت خام نسبت به روغن استخراج شده از كباب آماده شده افزايش نشان داد. افزايش عدد پراکسيد به طور عمده در مراحل نهايي پخت و به ميزان 127 تا 232 درصد از مقدار اوليه بود. در حالي که افزايش پراکسيد تابع زمان مواجهه با آتش پخت بود. تغييرات عدد اسيدي مستقل از روند زمان پخت گوشت و ازدياد عدد پراکسيد در آن بود.

Title: تحلیل و بررسی برخی از شاخص‌های جمعیت شناختی و بهداشتی شهرستان چادگان طی سال‌های 88–1383

http://hsr.mui.ac.ir/index.php/jhsr/article/view/442

مقدمه: برای بیان وضعیت موجود در یک منطقه، شاخص‌های جمعیتی و بهداشتی از اهمیت خاصی برخوردارند. هدف کلی این تحقیق تحلیل و بررسی برخی شاخص‌های جمعیتی از سال 1383 تا سال 1388 بود. روش‌ها: جامعه هدف در این تحقیق مردم شهرستان چادگان طی سال‌های 88-1383 بودند. اطلاعات جمعیتی خام که از مرکز بهداشت شهرستان جمع‌آوری شده بود، به عنوان معیار ورود در طی این سال‌ها مورد تحلیل دموگرافیک قرار گرفت. تحقیق از نوع توصیفی بود که به روش اسنادی انجام گردید. یافته‌ها: طبق نتایج تحقیق جمعیت شهرستان در طی این پنج سال کاهش چشم‌گیر داشته است. میزان تولدهای خام در سال 83، 2/14 در هزار و در سال 88، 4/15 در هزار بود. مرگ و میر خام در سال 83، 8/4 و در سال 88، 4/4 در هزار بود. میزان مرگ و میر زیر یک‌سال در سال83، 7/26 و در سال88، 7/16؛ مرگ و میر زیر پنج سال در سال 83، 4/29 و در سال 88، 2/18؛ درصد پوشش تنظیم خانواده در سال 83، 5/73 و در سال 88، 5/71؛ نسبت جنسی در سال 83، 5/98 و در سال 88، 2/99 و بالاخره میزان ناخالص تجدید نسل در سال 83، 34/0 و در سال 88، 35/0 در هزار بوده است. نتیجه‌گیری: کاهش رشد جمعیت به دلایل مختلفی از جمله مهاجرت، عوامل اقتصادی، اجتماعی، فرهنگی و بهداشتی، سیاست‌های جمعیتی و یا مجموعه‌ای از عوامل فوق‌الذکر بوده است. ضعف بهداشتی در منطقه و کمبود امکانات باعث گردیده است که میزان‌ مرگ و میر در حد بالایی نسبت به استان و کشور قرار داشته باشد. عدم ثبت دقیق و عدم دقت در اعلام گزارش نیز از مشکلات مربوط به آمارهای داده شده می‌باشد.

Title: تأثیر شوری بر گیاه پالایی کادمیوم از خاک‌های آلوده

http://hsr.mui.ac.ir/index.php/jhsr/article/view/444

مقدمه: گیاه پالایی یکی از روش‌های مورد استفاده در حذف آلاینده‌ها است که ضمن سادگی، ارزان قیمت و مؤثر بودن، باعث تخریب محیط زیست نمی‌شود. هدف این تحقیق، گیاه پالایی خاک آلوده به فلز سنگین کادمیوم و بررسی هم‌زمان تأثیر مقادیر مختلف هدایت الکتریکی و مقایسه توان اصلاح کننده‌های آلی لجن فاضلاب و کمپوست شهری برای بالابردن راندمان جذب فلز به وسیله گل آفتابگردان است. روش‌ها: آزمایش‌های این تحقیق در طرح اسپلیت پلات شامل 24 گلدان، به صورت تصادفی با سه تکرار در فاصله زمانی خرداد تا مرداد ماه 1389 انجام گردید. به منظور تعیین تأثیر شوری بر کارایی جذب فلز توسط گیاه آفتابگردان، از آب آبیاری با مقادیر مختلف هدایت الکتریکی (در دامنه 0، 2، 4 و dS/m 6) استفاده گردید. همچنین اثر ترکیبات اصلاح کننده آلی شامل لجن فاضلاب و کمپوست شهری برای بالابردن راندمان جذب فلز نیز مورد بررسی قرار گرفت. یافته‌ها: بیشترین مقدار جذب کادمیوم در هدایت الکتریکی dS/m 6 در بستر لجن، در ریشه 7/0 ± 8/5، در ساقه 6/3 ± 8/11 و در برگ mg/kg 0/0 ± 4/8 بود که باعث کاهش وزن خشک گیاه شد و بیشترین آن در تیمار کمپوست بود. نتیجه‌گیری: آنالیز واریانس داده‌ها نشان داد که با افزایش هدایت الکتریکی آب آبیاری، مقدار کادمیوم جذب شده در ریشه، ساقه و برگ گیاه افزایش می‌یابد. همچنین میزان جذب کادمیوم در تیمار لجن نسبت به تیمار کمپوست بیشتر بود. بنابراین کاربرد هدایت الکتریکی و لجن فاضلاب باعث افزایش میزان حذف کادمیوم می‌شود.

Title: بررسی کارایی گندزدایی کاهو بر اساس روش متداول در ایران

http://hsr.mui.ac.ir/index.php/jhsr/article/view/445

مقدمه: سبزیجات خام ممکن است حاوی انواع میکروارگانیسم‌ها و تخم انگل باشند و در صورتی که به شیوه مناسبی سالم‌سازی نگردند، باعث ابتلای انسان به انواع بیماری‌های انگلی و بیماری‌های عفونی روده‌ای می‌گردند. بنابراین استفاده از یک روش مؤثر در کاهش میکروارگانیسم‌های بیماری‌زا دارای اهمیت زیادی است. در اين تحقيق، کارايي دستورالعمل توصيه شده توسط وزارت بهداشت، درمان و آموزش پزشکی ایران جهت گندزدايي سبزيجات مورد ارزیابی قرار گرفت. این دستورالعمل شامل مراحل شست‌وشوی سبزیجات با آب، انگل‌زدایی سبزیجات (سه تا پنج قطره دترجنت در هر ليتر با زمان تماس 5 دقيقه) و گندزدايي سبزیجات (200 ميلي‌گرم در لیتر پرکلرین با زمان تماس 5 دقيقه) است. روش‌ها: بار اوليه ميکروبي (کل کليفرم و کليفرم مدفوعي) با استفاده از آزمايش MPN و بار انگلي موجود بر روي کاهو به روش شناورسازی تعيين گردید. نمونه کاهو بر اساس دستورالعمل توصیه شده وزارت بهداشت گندزدايي گرديد و پس از هر مرحله از گندزدايي بار ميکروبي و انگلي نمونه کاهو تعيين شد و در نهايت، کارايي گندزدايي بر اساس دستورالعمل پیش‌گفت مورد بررسي قرار گرفت. یافته‌‌ها: میانگین بار اوليه کل کليفرم و کليفرم مدفوعي موجود بر روي کاهو به ترتيب CFU/g 10log 36/3 و CFU/g 10log 31/2 بود. در هيچ کدام از نمونه‌هاي مورد آزمايش، آلودگي انگلي مشاهده نگردید. پس از گندزدايي کاهو بر اساس دستورالعمل توصیه شده وزارت بهداشت در مرحله شست‌وشو با آب، میزان حذف کل کليفرم و کلیفرم مدفوعی به ترتیب 1/78 درصد (CFU/g 10log 75/0) و 6/79 درصد (CFU/g 10log 67/0) و در مرحله استفاده از دترجنت میزان حذف کل کليفرم و کلیفرم مدفوعی به ترتیب 8/94 درصد (CFU/g 10log 44/1) ،5/98 درصد (CFU/g 10log 90/1) و در مرحله گندزدايي با پرکلرين کل کليفرم و کلیفرم مدفوعی به ترتیب 3/98 درصد (CFU/g 10log 18/2) و 100 درصد (CFU/g 10log 31/2) کاهش يافت. نتیجه‌گیری: دستورالعمل وزارت بهداشت به عنوان يک روش مؤثر در سالم‌سازی سبزیجات می‌تواند 3/98 درصد از کل کليفرم و 100 درصد از کليفرم مدفوعي موجود بر روی کاهو را حذف نمايد.واژه‌‌های کلیدی: سبزیجات خام، گندزدایی،کل کلیفرم، کلیفرم مدفوعی، وزارت بهداشت، کاهو.

Title: بررسی ارتباط شاخص نمره گذاری استرین گرمایی و شاخص دمای تر گوی سان با شاخص استرین فیزیولوژیکی در محیط کار گرم در مردان

http://hsr.mui.ac.ir/index.php/jhsr/article/view/577

مقدمه: استرس گرمایی یک مشکل معمول و همیشگی در بعضی صنایع می‌باشد. برای ارزیابی استرس گرمایی از شاخص‌های متعددی استفاده می‌شود که یکی از این شاخص ها،WBGT  می‌باشد؛ ولی این شاخص دارای محدودیت‌هایی نظیر هزینه و زمان پاسخ‌گویی بالا می‌باشد. لذا این شاخص یک ابزار غربالگری مناسب نیست. شاخص نمره‌گذاری استرین گرمایی (HSSI) نیز یک تکنیک مشاهده‌ای­قضاوتی است که جهت غربالگری اولیه تدوین شده است. هدف از این مطالعه، بررسی همبستگی شاخص HSSI با شاخص استرین فیزیولوژیکی (PSI) در شرایط نرمال است تا در صورت وجود همبستگی بالا بتوان از HSSI به عنوان یک ابزار غربالگری اولیه استرین گرمایی در محیط کار استفاده کرد. روش‌ها: این مطالعه یک مطالعه تحلیلی می‌باشد که بر روی 145 نفر نمونه مرد سالم انجام شد. ضربان قلب و دمای دهانی (جهت محاسبه شاخص PSI) با استفاده از دستگاه ضربان‌سنج قلب (مدل POLAR 100 RS) و دماسنج دهانی (مدل 100(TB  در حین استراحت و سپس به مدت 5/1 ساعت در حین فعالیت کاری ثبت شد و به طور همزمان شاخص WBGT ثبت و پرسش‌نامه شاخص HSSI نیز تکمیل شد. در نهایت با استفاده از آزمون همبستگی Pearson و Regression analysis، همبستگی این سه شاخص بررسی شد. یافته‌ها: آزمون همبستگی Pearson بین شاخص HSSI و PSI ارتباط مستقیم و معنی‌داری را نشان داد (001/0 > P و 57/0 = r). بین شاخص  WBGTو PSI نیز ارتباط مستقیم معنی‌داری مشاهده شد (001/0 > P و 397/0 = r) و همچنین بین شاخص HSSI و دمای دهانی نیز همبستگی بالایی مشاهده شد (556/0 = r). نتیجه‌گیری: شاخص HSSI در مقایسه با شاخص WBGT همبستگی بالاتری با شاخص PSI نشان داد، همچنین مقادیر شاخص‌های WBGT، PSI و دمای دهانی برای سطوح ریسک شاخص HSSI روندی افزایشی داشت. در نتیجه با توجه به همبستگی بالاتر شاخص HSSI و مزایای دیگر آن، این شاخص می‌تواند به عنوان یک ابزار غربالگری اولیه استرین گرمایی در محیط‌های کاری ایران استفاده شود.

Title: تحلیل محتوای مطالب بهداشتی روزنامه‌های کثیر الانتشار ایران با تأکید بر ارتقای سلامت و آموزش بهداشت در سال 1386

http://hsr.mui.ac.ir/index.php/jhsr/article/view/454

مقدمه: برای رسیدن به هدف سلامتی بهتر، هر جامعه‌ای نیازمند همکاری بخش‌های مختلف اجتماعی است، که به نحوی بر افکار عمومی و سیاستگذاران تأثیرگذار می‌باشد. این تحقیق با هدف تحلیل محتوای مطالب بهداشتی روزنامه‌های کثیر الانتشار ایران با تأکید بر ارتقای سلامت و آموزش بهداشت در سال 1386 انجام شد. روش‌ها: در این پژوهش از روش تحلیل محتوا استفاده شد. نمونه‌ها شامل دو هفته آماری از ده روزنامه پرخواننده ایران و در مجموع 114 شماره بود. پس از جمع‌آوری و دسته‌بندی مطالب بهداشتی روزنامه‌های مورد نظر داده‌ها وارد نرم‌افزار SPSS شد و به وسیله آزمون 2χ در سطح معنی‌داری آنالیز گردید (05/0 P <). یافته‌ها: تعداد 12 شماره روزنامه فاقد هر گونه مطلب بهداشتی و بقیه روزنامه‌ها 495 مطلب بهداشتی داشتند. نتایج نشان داد که 57/2 درصد از سطح روزنامه‌های مورد بررسی به مطلب بهداشتی اختصاصی داشت. مطالب بهداشتی در روزنامه‌های کشور برجسته‌سازی نمی‌شود؛ به طوری که فقط 7/7 درصد از مطالب بهداشتی در صفحات مهم روزنامه‌ها چاپ شده بودند. همچنین 41 درصد از مطالب دارای تیتر چهارم و 7/58 درصد از مطالب فاقد عکس بودند. موضوع بیماری‌ها با 7/44 درصد، بیش‌ترین موضوع مطالب را به خود اختصاص داد. بحث و نتیجه‌گیری: با توجه به حجم کم مطالب بهداشتی و عدم برجسته‌سازی موضوع سلامت در روزنامه‌ها، به نظر می‌رسد که تلاش برای آشناسازی متخصصین بهداشت با توانایی‌های رسانه‌ها و استفاده از آن‌ها برای ارتقای سلامت و ایجاد رویکردی جدید در برنامه‌های رسانه‌ها برای پرداختن به موضوع سلامت از اهمیت زیادی برخوردار باشد.

Title: سبک‌هاي يادگيري دانشجويان بهداشت محیط و تغذیه دانشگاه علوم پزشکی اصفهان

http://hsr.mui.ac.ir/index.php/jhsr/article/view/455

مقدمه: «سبک يادگيري» روشي است که يادگيرنده در يادگيري به روش‌هاي ديگر ترجيح مي‌دهد. اين پژوهش با هدف شناخت سبک‌هاي يادگيري دانشجويان رشته بهداشت محيط ترم 2 و 4 و نیزدانشجویان رشته تغذيه ترم‌هاي 1، 2، 3‌ و 4 دانشکده بهداشت دانشگاه علوم پزشکی اصفهان انجام شد. روش‌ها: در این مطالعه مقطعي- تحليلي دانشجويان بهداشت محيط ترم 2 و 4 و دانشجويان تغذيه ترم 1، 2، 3 و 4 دانشکده بهداشت دانشگاه علوم پزشکي اصفهان با استفاده از سرشماری انتخاب شدند. پرسش‌نامه سياهه سبک‌هاي يادگيري (LSI) تهيه شده توسط ديويد کلب برای تعیین سبک‌های یادگیری در اختیار دانشجویان قرار داده شد. تجزيه و تحليل داده‌ها با استفاده از شاخص‌هاي آماري توصيفي با استفاده از نرم‌افزار SPSS صورت گرفت. یافته‌ها‌: تعداد کل دانشجویان این دو رشته 170 نفر بود. افرادی که در مطالعه شرکت کردند 134 نفر بودند که سه نفر از آن‌ها به خاطر اینکه جزء گروه مورد مطالعه نبودند، از مطالعه حذف شدند. از 131 نفر باقی‌مانده، 76 نفر در رشته تغذیه و 55 نفر در رشته بهداشت محیط مشغول تحصیل بودند. 29 نفر از جامعه پژوهش مرد و 102 نفر زن بودند. سبک غالب یادگیری در مردان جذب کننده و در زنان هم‌گرا بود. فراوانی سبک‌های یادگیری واگرا، تطابق یابنده، هم‌گرا و جذب کننده در رشته تغذیه به ترتیب 18 (7/23 درصد)، 18 (7/23 درصد)، 18 (7/23 درصد) و 22 (9/28 درصد) و فراوانی سبک‌های یادگیری واگرا، انطباق یابنده، هم‌گرا وجذب کننده در رشته بهداشت محیط به ترتیب 16 (1/29 درصد)، 17 (9/30 درصد)، 13 (6/23 درصد) و 9 (4/16 درصد) بود. نتيجه‌گيري: دانشجویان رشته‌های بهداشت محیط و تغذیه، سبک‌های غالب یادگیری مختلفی دارند.

Title: ارزیابی کیفیت زندگی بیماران مبتلا به نارسایی کلیه تحت همودیالیز و پیوند کلیه در مقایسه با افراد سالم

http://hsr.mui.ac.ir/index.php/jhsr/article/view/456

مقدمه: نارسايي کلیوی در سراسر دنیا یکی از مشکلات عمده سلامت عمومی محسوب می‌شود. ميزان شيوع این بیماری بالا است و ساليانه حدود 8 درصد به اين ميزان افزوده مي‌شود. با وجود تلاش‌هاي بسيار به منظور پيشگيري از بيماري انتهايي كليه، تعداد اين بيماران در كشور در حال افزايش است. بروز بيماري نارسايي مزمن كليوي و انجام روش‌هاي درماني از جمله همودياليز و پيوند كليه منجر به تغيير در شيوه زندگي، وضعيت سلامت و ايفاي نقش اجتماعي فرد می‌شود كه همه اين عوامل به نوعي كيفيت زندگي بيماران را تحت تأثير قرار مي‌دهد. مطالعه حاضر با هدف تعیین و مقایسه کیفیت زندگی بیماران مبتلا به نارسایی کلیه تحت همودیالیز و پیوند کلیه در مقایسه با افراد سالم انجام گردید. روش‌ها: مطالعه حاضر از نوع مطالعات مورد- شاهدی بود كه در آن متغير كيفيت زندگي، در دو گروه بيماران مبتلا به نارسايي مزمن كليوي تحت درمان با همودياليز و پيوند كليه مراجعه کننده به بیمارستان‌های تابعه دانشگاه علوم پزشکی تهران مورد سنجش قرار گرفت و گروه شاهد شامل 200 نفر از افراد سالم بودند. ابزار گردآوري داده‌ها در این مطالعه، از دو بخش متغیرهای فردی و مرتبط با بیماری و پرسش‌نامه استاندارد کیفیت زندگی (WHOQOL-BREF) تشکیل مي‌شد. داده‌ها پس از جمع‌آوري با استفاده از نرم‌افزار 18SPSS و با استفاده از آمار توصیفی و استنباطی نظیر آزمون همبستگی پیرسون و آنالیز واریانس مورد تجزیه و تحلیل قرار گرفت. یافته‌ها: میانگین سنی بیماران همودیالیزی 93/16 ± 25/51 سال بود، بیماران پیوندی 52/12 ± 56/38 سال و افراد سالم 12 ± 83/38 سال داشتند که  تفاوت آماری معنی‌داری بین سه گروه وجود داشت (05/0 P ≤). از نظر جنس و وضع تأهل، تفاوت معنی‌داری مشاهده نشد (05/0 ≥ P)؛ اما از نظر تحصيلات و وضعيت اقتصادي ما بين سه گروه تفاوت معنی‌داري وجود داشت (05/0 ≤ P). میانگین نمره کیفیت زندگی بیماران همودیالیزی80/11 ± 41/42 و پیوند کلیه80/7 ± 03/66 و افراد سالم 36/9 ± 42/56 بود که از نظر آماری ارتباط معنی‌داری مشاهده شد (05/0 ≤ P). بيشترين نمره كيفيت زندگي مربوط به بيماران پيوند كليه و كمترين نمره مربوط به بيماران همودياليزي بود. نتیجه‌گیری: نتایج به دست آمده از اين مطالعه نشان داد كه چگونه بيماري انتهايي كليه (ESRD) و درمان‌هاي جايگزين، عملکرد اجتماعی، سلامت جسمی، روانی و محیطی بيماران را تحت تأثير قرار داده بود. بر اساس نتايج پژوهش حاضر، بيماران پيوند كليوي نمره كيفيت زندگي بالاتري را نسبت به بیماران همودیالیزی كسب نمودند كه اين نشان مي‌دهد بيماران پيوند كليوي قادر به گذراندن زندگي بهتري بعد از عمل پيوند كليه هستند. به گونه‌اي كه در اين مطالعه، بيماران پيوند كليه كيفيت زندگي خود را بالاتر از افراد سالم گزارش نموده‌اند. به نظر مي‌رسد يك پيوند كليه موفق، تأثير مثبتي بر درك وضعیت سلامتي دارد و چهار دامنه كيفيت زندگي مرتبط با سلامت را بهبود می‌بخشد و به واسطه بالا بردن عزت نفس، حس آزادي را به همراه مي‌آورد.

Title: بررسي اضطراب امتحان و ارتباط آن با عملكرد تحصيلي دانشجويان مامايي

http://hsr.mui.ac.ir/index.php/jhsr/article/view/457

مقدمه: اضطراب يكي از احساسات عاطفي انسان است. اما هنگامي كه این احساس شدت مي‌یابد، ممكن است به نتايج نامطلوبی منجر گردد. يكي از بيشترين وقايع تهديد كننده كه امروزه موجب اضطراب در دانشجويان مي‌گردد، امتحانات است. اين مطالعه به بررسي ارتباط اضطراب امتحان و عملكرد تحصيلي دانشجويان مامايي دانشگاه آزاد اسلامي واحد نجف‌آباد پرداخته است. روش‌ها: مطالعه حاضر يک مطالعه مقطعي به صورت توصيفي- تحليلي بود كه بر روي 114 نفر از دانشجويان مامايي مشغول به تحصيل در سال 1390 انجام شد. ابزار گردآوري داده‌ها شامل دو پرسش‌نامه زمينه‌اي 14 سؤالي و تست اضطراب امتحان ساراسون 37 سؤالي بود. پرسش‌نامه طي مدت يك هفته، در اواخر ارديبهشت ماه در بين شرکت کنندگان توزيع شد. تجزيه و تحليل داده‌ها با استفاده از آزمون t زوج و t مستقل، آزمون Chi-square، ضریب همبستگی Pearson و Spearman و آنالیز واریانس در سطح معني‌دار 05/0 توسط نرم‌افزار 16SPSS انجام شد. يافته‌ها: در اين پژوهش، ميانگين نمره اضطراب امتحان دانشجويان 08/17 بود. اضطراب امتحان در 33 نفر از دانشجويان (9/28 درصد) در حد كم، در 51 نفر (7/44 درصد) در حد متوسط و در 30 نفر (3/26 درصد) شديد بود. بين اضطراب امتحان و عملكرد تحصيلي دانشجويان مامايي ارتباط معنی‌دار آماري (044/0 = P، 189/0- = r) وجود داشت. نتيجه‌گيري: با توجه به زياد بودن اضطراب امتحان در دانشجويان مامايي و ارتباط معكوس و معنی‌دار آن با عملكرد تحصيلي، كمك به يادگيري دانشجويان براي مديريت مؤثر اضطراب، يك وظيفه چالش‌انگيز است كه نياز به تلاش گروهي دارد.

Title: بررسی ارتباط استرس و ابعاد مختلف سبک زندگي با کيفيت زندگي در افراد 19 سال و بالاتر اصفهان و حومه، کاربرد مدل رگرسيون کلاس پنهان با متغيرهاي پيش‌بين پنهان

http://hsr.mui.ac.ir/index.php/jhsr/article/view/458

مقدمه: بررسي کيفيت زندگي و عوامل اثرگذار بر آن در جمعيت‌هاي عمومي باعث به وجود آمدن يک ديدگاه کلي از وضعيت سلامت جامعه و ارایه راهکارهای مربوط به سیاست‌گزاری و برنامه‌ریزی‌های بهداشتی در جامعه می‌شود. تحقيق حاضر با هدف بررسي رابطه استرس و مؤلفه‌های مهم سبک زندگی، یعنی فعاليت فيزيکي و عملکرد تغذيه‌اي بر کيفيت زندگي، با کنترل اثر مجموعه مهمی از متغيرهاي زمينه‌اي، در جمعيت عمومي 19 سال و بالاتر ساکن اصفهان و حومه انجام شده است. روش‌ها: در یک مطالعه مقطعی بر مبنای نتایج طرح "برنامه ملی قلب سالم اصفهان" در سال 1387، تعداد 4619 نفر از افراد 19سال و بالاتر به روش نمونه‌گیری تصادفی خوشه‌ای چند مرحله‌ای انتخاب شدند و مورد بررسی قرار گرفتند. پرسش‌نامه کیفیت زندگی (World health organization quality of life-Bref یا WHOQOL-Bref)، پرسش‌نامه سنجش استرس (General health questionnaire-12 یا GHQ-12)، پرسش‌نامه ارزیابی فعالیت فیزیکی Baecke، پرسش‌نامه ارزیابی عملکرد تغذیه‌ای FFQ (Food frequency questionnaire) و اطلاعات جمعیت‌شناختی مورد استفاده قرار گرفت. تحلیل داده‌ها با استفاده از مدل رگرسیون کلاس پنهان با متغیرهای پیش‌بین پنهان انجام گردید. یافته‌ها: حدود 48 درصد شرکت‌کنندگان در کلاس کیفیت زندگی متوسط قرار گرفتند، 29 و 23 درصد آن‌ها به ترتیب در کلاس‌های با کیفیت زندگی پایین و خوب قرار گرفتند. بانفوذترین متغیرهای پیش‌بین در متمایز کردن افراد در کلاس‌های مختلف کیفیت زندگی عبارت از فعالیت فیزیکی، وضعیت مصرف دخانیات، وضعیت اقتصادی، جنسیت، استرس و ابتلا به عوامل خطر بیماری‌های قلبی- عروقی بودند. متغیرهای وضعیت تأهل و محل سکونت فاقد اثر معنی‌دار آماری بودند. نتیجه‌گیری: افزایش سطح آگاهی عمومی به منظور تغییر و تعدیل عوامل ناسالم مرتبط با سبک زندگی و آموزش شیوه‌های مقابله با استرس و زمینه‌سازی برای برخورداری مناسب از مراقبت‌های بهداشتی (به ویژه با توجه به اهمیت نقش سطح درآمد) از طریق توزیع متوازن ثروت در جامعه، موجب ارتقای کیفیت زندگی افراد در جامعه می‌شود و در نهایت باعث افزایش بهره‌وری نیروی انسانی و تعالی ساختار انسانی و اجتماعی جامعه می‌گردد. چنین مداخلاتی می‌بایست متناسب با ویژگی‌های طبقات جنسیتی، سنی و تحصیلی برنامه‌ریزی و اجرا گردند.

Title: حذف اتیل بنزن و زایلن از محلول‌های آبی توسط نانولوله‌های کربنی

http://hsr.mui.ac.ir/index.php/jhsr/article/view/459

مقدمه: حذف اتيل بنزن و زايلن از محلول‌هاي آبي با استفاده از نانولوله‌هاي کربني تک ديواره (SWCNT یا Single walled carbon nano tubes)، نانولوله‌هاي کربني چند ديواره (MWCNT یا Multi walled carbon nano tubes) و نانولوله‌هاي کربني هيبريد (HCNT یا Hybrid carbon nano tube) مورد بررسي قرار گرفت. روش‌ها: نمونه‌هاي مورد نياز به صورت سنتتيک با غلظت mg/l 10 از اتيل بنزن و زايلن، غلظت g/l 1 نانولوله‌هاي کربني و زمان تماس 10 دقيقه در pH خنثي توسط دستگاه گاز کروماتوگرافي آناليز شد. براي آناليز نمونه‌ها از نرم‌افزار طراحي آزمايش (DOE یا Design of experiment) استفاده شد. یافته‌ها: ظرفيت جذب اتيل بنزن و زايلن توسط SWCNT بيشتر از MWCNT و HCNT (براي اتيل بنزن mg/g 97/9 و براي زايلن mg/g 10 توسط SWCNT) بود. زايلن بيشتر از اتيل بنزن جذب شد که ناشي از بيشتر بودن قابليت انحلال اتيل بنزن و کمتر بودن وزن مولکولي آن نسبت به زايلن است. بازيابي نانولوله‌هاي استفاده شده با استفاده از گرما در دماي ºC2 ± 105 براي اولين بار در اين تحقيق انجام گرفت، که علاوه بر بازيابي باعث بهبود عملکرد آن‌ها در حذف اتيل بنزن نسبت به حالت اوليه نيز گرديد. نتیجه‌گیری: SWCNT توانست به طور مؤثري اتيل بنزن و زايلن را از آب جذب نمايد و داراي پتانسيل خوبي براي تصفيه آب و فاضلاب‌هاي آلوده به مواد نفتي بود.

Title: بررسی ارتباط حمایت اجتماعی درک شده با سلامت اجتماعی سالمندان شهر اصفهان

http://hsr.mui.ac.ir/index.php/jhsr/article/view/461

مقدمه: در قرن حاضر افزايش سريع تعداد سالمندان، وجود ناتواني، كاهش استقلال و افزايش وابستگي در بسیاری از آنان، ضرورت توجه به مسأله حمایت اجتماعی را در این قشر افزايش داده است. حمایت اجتماعی بر ابعاد مختلف سلامت سالمندان تأثیرگذار است. یکی از ابعاد مهم سلامت که کمتر در این گروه مورد توجه قرار گرفته است، سلامت اجتماعی آنان می‌باشد. سلامت اجتماعی به میزانی از عملکرد، تطابق و سازگاری در زندگی اجتماعی اطلاق می‌شود. لذا هدف از انجام این مطالعه تعیین ارتباط بین حمایت اجتماعی درک شده با وضعیت سلامت اجتماعی سالمندان شهر اصفهان بوده است. روش‌ها: اين مطالعه توصیفی- تحلیلی و از نوع مقطعی بر روي 230 سالمند ٦٠ سال به بالای شهر اصفهان انجام شد. نمونه‌ها به روش تصادفی ساده از سطح شهر انتخاب شدند. داده‌ها توسط پرسش‌نامه ویژگی‌های دموگرافیک، مقیاس چند بعدی حمایت اجتماعی درک شده و مقیاس سلامت اجتماعی Keyes جمع‌آوری شد. سرانجام نمره نهایی کسب شده از این مقیاس‌ها در سه دسته خوب، متوسط و ضعیف (در طیف نمره 100-0) گزارش گردید. اطلاعات گردآوري شده توسط آزمون‌هاي همبستگي Pearson و Spearman، ANOVA و Independent t-test مورد تجزيه و تحليل قرار گرفتند. یافته‌ها: 9/70 افراد مورد مطالعه مرد بودند. میانگین سنی افراد مورد مطالعه 1/7 ± 46/67 بوده است. میانگین نمره کلی حمایت اجتماعی درک شده و سلامت اجتماعی سالمندان به ترتیب 8/28 ± 16/71 و 3/ 18 ± 98/50 بود. بین نمره حمایت اجتماعی درک شده با نمره سلامت اجتماعی ارتباط مستقیم و معنی‌دار وجود داشت (001/0 > P). بین برخی از متغیرهای دموگرافیک افراد و نمرات حمایت اجتماعی درک شده و سلامت اجتماعی ارتباط مستقیم و معنی‌داری مشاهده گردید (001/0 > P). نتيجه‌گيري: با توجه به تأثیرگذاری مقوله حمایت اجتماعی درک شده بر سلامت اجتماعی سالمندان و اهمیت این جنبه از سلامت، ضرورت توجه بیشتر به این موضوع در قشر آسیب‌پذیر سالمند احساس می‌گردد.

Title: پیش‌بینی عوامل مؤثر بر انجام تست پاپ اسمیر در بین زنان با بهره‌گیری از مدل اعتقاد بهداشتی

http://hsr.mui.ac.ir/index.php/jhsr/article/view/463

مقدمه: سرطان دهانه رحم پس از سرطان‌هاي پوست، پستان و ريه، شايع‌ترين سرطان در زنان مي‌باشد و در کل سرطان سرويكس دومين سرطان شايع در تمام دنيا است. با توجه به اهمیت موضوع در مطالعه حاضر به تعيين عوامل پيش‌بيني كننده انجام تست پاپ اسمیر در بین زنان متأهل مراجعه کننده به مراکز بهداشتی- درمانی شهر همدان با بهره‌گيري از مدل اعتقاد بهداشتی پرداخته شده است. روش‌ها: این پژوهش یک مطالعه توصيفي بود که به روش مقطعي بر روي 400 نفر از زنان متأهل مراجعه کننده به مراکز بهداشتی- درمانی شهر همدان انجام شد. نمونه‌گيري به صورت طبقه‌بندی تصادفی ساده صورت گرفت. ابزار مورد استفاده جهت جمع‌آوری اطلاعات، پرسش‌نامه استاندارد بود. در نهایت داده‌ها‌ جمع‌آوری گردید و در نرم‌افزار آماری 13SPSS وارد شد و مورد تجزیه و تحلیل قرار گرفت. یافته‌ها: 8/63 درصد از زنان سابقه انجام پاپ اسمیر را داشتند و 3/28 درصد از آنان نیز گزارش کرده بودند که تست پاپ اسمیر را به طور منظم انجام می‌دهند. بين سابقه وجود فرد مبتلا به سرطان دهانه رحم در خانواده، سن و انجام منظم تست پاپ اسمیر رابطه آماری معنی‌داری وجود داشت (05/0 > P). همچنین آزمون آنالیز رگرسیون لوجستیک نشان داد که موانع درک شده، پیش‌بینی کننده قوی در انجام تست پاپ اسمیر به طور منظم در بین زنان مورد بررسی بوده است. نتیجه‌گیری: متخصصین مراقبت از سلامتی زنان باید اطلاعات مناسبی در مورد عوامل مرتبط با سرطان دهانه رحم داشته باشند. با توجه به نتایج، به نظر می‌رسد که طراحی استراتژی‌های مناسب به منظور کاهش موانع درک شده تست پاپ اسمیر در بین زنان بتواند نتایج مفیدی را در این زمینه به دنبال داشته باشد.

Title: جنبه‌های اپیدمیولوژیک، بالینی و آزمایشگاهی تب مالت در خرم‌آباد

http://hsr.mui.ac.ir/index.php/jhsr/article/view/508

مقدمه: بروسلوز به عنوان یک بیماری مشترک بین انسان و حیوان، هنوز هم یک مشکل بهداشت عمومی در بسیاری از کشورها از جمله ایران است و برای انجام پیش‌گیری، کنترل و سیاست ریشه‌کنی این بیماری، نیاز به اطلاعات دقیق و الگوهای اپیدمیولوژیک آن در کشور می‌باشد. این مطالعه با هدف تعیین جنبه‌های اپیدمیولوژیکی، بالینی و آزمایشگاهی تب مالت در بیماران دارای سابقه بستری انجام شد.   روش‌ها: اين پژوهش يك مطالعه مقطعي بود كه بر روي 126 بیمار مبتلا به تب مالت بستری شده در شهرستان خرم‌آباد (بیمارستان شهدای عشایر) در طي سال‌هاي 84 الی 89 انجام شد. داده‌ها از پرونده بیماران جمع‌آوری و پس از وارد كردن كليه داده‌ها به نرم‌افزار SPSS نسخه 16 با استفاده از شاخص‌هاي آمار توصيفي و 2χ، آنالیز واریانس یک طرفه و t مستقل مورد تجزيه و تحليل آماري قرار گرفت.   یافته‌ها: 9/57 درصد (73 نفر) از افراد بیمار از جنس مرد و 73 درصد (92 نفر) از افراد در روستا سکونت داشتند و از نظر ميانگين سني بين بیماران شهري و روستايي تفاوت معنی‌داری وجود داشت. در این مطالعه 7/37 درصد سابقه مصرف لبنیات غیر پاستوریزه مثبت اعلام شده بود. فراوانی تشخیص اولیه در مناطق شهری و روستایی تفاوت معنی‌داری داشت و بیشترین رژیم درمانی مورد استفاده، ترکیب دارویی ریفامپین و داکسی سایکلین بود.   نتیجه‌گیری: كنترل بيماري تب مالت عمدتاً بر پايه پيش‌گيري قرار دارد و با توجه به بالا بودن بروز بيماري در مردان و زنان روستایی، آموزش براي پيش‌گيري از بيماري در هنگام تماس با دام‌ها و مصرف لبنیات غیر پاستوریزه مي‌تواند در كنترل بيماري كمك كننده باشد.

Title: بررسی ارتباط میزان استرس شغلی با غیبت‌های ناشی از کار در پرسنل اداري مرکز بهداشت استان اصفهان

http://hsr.mui.ac.ir/index.php/jhsr/article/view/466

مقدمه: استرس شغلی زمانی رخ می‌دهد که نیازهای شغلی با توانایی‌ها حمایت‌ها و نیازهای فرد شاغل همخوانی نداشته باشد. استرس شغلی علاوه بر این که می‌تواند بر سلامت جسمی و روانی افراد تأثیر منفی بگذارد، باعث کاهش کارایی آن‌ها شده، بهره‌وری را در سازمان‌ها کاهش دهد. از این رو این مطالعه با هدف بررسي ارتباط میزان نمره استرس شغلی با غیبت‌های ناشی از کار در پرسنل اداري مرکز بهداشت استان اصفهان انجام شد. روش‌ها: این مطالعه توصیفی- تحلیلی از نوع مقطعی بود كه 82 نفر از پرسنل مركز بهداشت استان اصفهان به طور كاملاً تصادفي وارد مطالعه شدند. ابزار گردآوري داده‌ها پرسش‌نامه شامل اطلاعات دموگرافيك به همراه پرسش‌نامه استرس شغلي Davis و همكاران با ضريب روايي همزمان (روايي ملاك) معادل 701/0 و ضريب پايايي به شيوه Cronbach’s alpha معادل 863/0 استفاده شد. حداقل نمره استرس صفر و حداکثر نمره 48 بود. اطلاعات از طریق نرم‌افزار SPSS با استفاده از آزمون‌های همبستگی Pearson، همبستگی Spearman، t مستقل و آنالیز واریانس مورد تجزیه و تحلیل قرار گرفت، و نتایج اعلام شد. يافته‌ها در اين بررسي 51 نفر (2/62 درصد) از افراد تحت مطالعه زن و 31 نفر (8/37 درصد) مرد بودند. میانگین و انحراف معیار نمره استرس 04/12 ± 05/19 به دست آمد. میانگین و انحراف معیار تعداد روزهای غیبت 6/7 ± 7/2 بود. آزمون همبستگی Pearson نشان داد که رابطه معنی‌داری بین نمره استرس و غیبت ناشی از کار وجود ندارد (122/0 = r، 141/0 = P). میانگین نمره استرس در خانم‌ها 45/12 ± 80/20 و در مردان 91/10 ± 16/16 است. آزمون t مستقل نشان داد که بین نمره استرس و جنس رابطه معنی‌داری وجود دارد، به این ترتیب که نمره استرس در خانم‌ها بیشتر از آقایان بود (046/0 = P). همچنین آزمون همبستگی Pearson نشان داد كه بین نمره استرس و درآمد رابطه معكوس وجود دارد (221/0- = r، 023/0 = P). بحث و نتيجه‌گيري نتایج مربوط به نمره استرس شغلی در پرسنل اداری مركز بهداشت استان اصفهان نشان داد که 7/20 درصد از افراد تحت مطالعه از استرس شغلي رنج مي‌برند. با توجه به نمره استرس شغلی در کارکنان برای سازمان ضروری است که با به کارگیری راه‌کارهای حمایتی از تأثیرات درازمدت استرس شغلی بر کارکنان پیش‌گیری کنند.

Title: سطح ویتامین ث (آسکوربیک اسید) در پلاسمای مادر و نوزاد و ارتباط آن با وزن تولد نوزاد

http://hsr.mui.ac.ir/index.php/jhsr/article/view/509

مقدمه: آسکوربیک اسید پلاسما یک شاخص منطقی در گزارشاتی است که پیشنهاد می‌کنند کاهش سطح اسکوربیک اسید مادر با عوارض خاصی مثل نارسی نوزاد، پره اکلامپسی، وزن کم تولد ((LBW و افزایش مرگ و میر نوزادی همراه است. هدف از این مطالعه تعیین سطوح اسکوربیک اسید در مادران و نوازدانشان و ارتباط آن با وزن تولد نوزاد بود. روش: 101 مادر مراجعه کننده به 5 زایشگاه مختلف شیراز جهت زایمان و نوزادان آن‌ها مورد بررسی قرار گرفتند. ویژگی‌های انتروپومتریک آن‌ها ثبت شده، نمونه خون مادران قبل از زایمان و بند ناف پس از تولد نوزاد جمع‌آوری می‌شد. آسکوربیک اسید پلاسما به روش رنگ‌سنجی اندازه‌گیری شده، اطلاعات جمع‌آوری شده به وسیله نرم‌افزار spss تجزیه و تحلیل شد. یافته‌ها: میانگین وزن تولد نوزادان 43/411 ± 87/3232 گرم و میانگین سن مادران 975/9 ± 604/23 سال بود. میانگین آسکوربیک اسید پلاسمای مادران 6/0 ± 229/1 میلی‌گرم در دسی‌لیتر و به طور معنی‌داری کمتر از مقدار آن در خون بند ناف (6/0 ± 974/1) بود (001/0 > P). آسکوربیک اسید پلاسما با وزن تولد رابطه مستقیم و معنی‌داری داشته، در گروه‌های وزنی کمتر از 3215 گرم اختلاف معنی‌داری را نشان داد (0006/0 = P). بحث سطوح بالاتر آسکوربیک اسید در بند ناف تئوری احتباس انتخابی را تأیید می‌کند، از سوی دیگر ویتامین ث رژیم غذایی برای حفظ سطح مناسب پلاسمایی به ویژه طی بارداری ضروری است که خود نوزاد را برای داشتن سطح مطلوب سلامتی و وزن تولد مناسب حمایت می‌کند

Title: بررسی کمی و کیفی زباله‌های دندان‌پزشکی شهر قزوین

http://hsr.mui.ac.ir/index.php/jhsr/article/view/510

مقدمه: با توجه به این که اولین قدم در مدیریت مواد زاید دندان‌پزشکی شناسایی و طبقه‌بندی این مواد می‌باشد، از این رو در این مطالعه، زباله‌های دندان‌پزشکی شهر قزوین از نظر کمی و کیفی در دو بخش خصوصی و دولتی در سه ماهه اول سال 2009 مورد بررسی قرار گرفت. روش‌ها: به منظور تحقق اهداف این مطالعه، مراکز دندان‌پزشکی موجود در شهر قزوین به دو گروه عمومی و خصوصی تقسیم‌بندی شد و از بین 32 مرکز عمومی و 98 مرکز خصوصی تعداد 10 مرکز دولتی و 40 مرکز خصوصی به صورت تصادفی انتخاب شد. با توجه به مطالعات انجام شده قبلی در زمینه زباله‌های دندان‌پزشکی، این مواد به 6 گروه (وسایل آلوده، نسج بیمار، وسایل نوک‌تیز و برنده، مواد مصرفی، مواد شیمیایی و مواد دندان‌پزشکی) تقسیم‌بندی شد. سپس اطلاعات به دست آمده توسط نرم‌افزار Excel مورد تجزیه و تحلیل قرار گرفت. یافته‌ها: تجزیه و تحلیل‌های انجام شده نشان می‌دهد که در مراکز خصوصی 12 درصد از زایدات تولیدی مربوط به وسایل آلوده، 1 درصد مربوط به نسج بیمار، 36 درصد مربوط به وسایل نوک‌تیز و برنده، 51 درصد مربوط به وسایل مصرفی و مقدار نسبي مواد دندان‌پزشکی و مواد شیمیایی بسیار ناچیز می‌باشد. در مراکز دولتی 13 درصد مواد زاید مربوط به وسایل آلوده، 8 درصد مربوط به نسج بیمار، 13 درصد مربوط به وسایل نوک‌تیز و برنده، 66 درصد مربوط به وسایل مصرفی و مقدار مواد دندان‌پزشکی و مواد شیمیایی صفر بود. نتیجه‌گیری: با توجه به نتایج به دست آمده، مشخص شد که بین وضعيت كمي و كيفي زباله‌های دندان‌پزشکی در مراکز خصوصی و عمومی رابطه معنی‌دار وجود دارد. از این رو به منظور بهبود روند مدیریت پسماندهای بیمارستانی شهر قزوین جهت مدیریت زباله‌های دندان‌پزشکی بایستی تمهیدات لازم از سوی دانشگاه علوم پزشکی، سازمان‌های بازیافت و شهرداری قزوین به منظور جمع‌آوری و دفع این مواد به صورت مجزا در نظر گرفته شود.

Title: بررسی علل تغذیه با شیر مصنوعی در مادران دارای كودك تك‌قلو و چند‌قلو تحت پوشش مركز شیر مصنوعی شهرستان لنجان

http://hsr.mui.ac.ir/index.php/jhsr/article/view/511

مقدمه: تغذیه با شیر مادر یك اصل مهم در تأمین سلامت كودكان در طی سال اول زندگی می‌باشد و مزایای آن برای كودك، مادر، خانواده و اجتماع مورد تأكید همه صاحب‌نظران می‌باشد. از این رو ضروری است که علل تغذیه با شیر مصنوعی مورد بررسی قرار گیرد و علل محروم شدن شیرخوار از نعمت شیر مادر تعیین شود. مواد و روش: این پژوهش یك مطالعه توصیفی تحلیلی است. داده‌ها از 144 مادر مراجعه كننده به مركز شیر مصنوعی شهرستان لنجان جمع‌آوری شد. یك كارشناس آموزش دیده طی 2 ماه از طریق بررسی پرونده خانوار و مصاحبه ساختار یافته با مادران به جمع‌آوری داده‌ها پرداخت و داده‌ها در  SPSSتجزیه و تحلیل شد. یافته‌ها: مهم‌ترین علت شروع شیر مصنوعی در مادران تك‌قلوزا، نگرفتن سینه (8/39 درصد) و در مادران چندقلوزا، دوقلویی و سه‌قلویی (13/89 درصد) بود. 08/54 درصد از مادران تك‌قلوزا یك علت و 74/71 درصد از مادران چندقلوزا بیش از یك علت را برای شروع شیر مصنوعی مطرح نموده بودند كه از لحاظ آماری معنی‌دار بود (004/0 = P). 33/57 درصد از مادران تك‌قلوزا بچه‌ها‌یشان را در 2ساعت اول پس از زایمان و 37/62 درصد از مادران چندقلوزا بچه‌ها‌یشان را در بیش از 2 ساعت اول پس از زایمان با شیر خود تغذیه كرده بودند كه از لحاظ آماری معنی‌دار بود (004/0 = P). زایمان سزارین در مادران چندقلوزا 83/97 درصد و در مادران تك‌قلوزا 49/74 درصد بود كه از نظر آماری معنی‌دار بود (001/0 = P). 26/25 درصد از كودكان چند‌قلو نارس؛ در حالی كه فقط 34/11 درصد از كودكان تك‌قلو نارس بودند و از لحاظ آماری معنی‌دار بود (012/0 = P). بحث: با توجه به این كه در این پژوهش درصد بالایی از مشكلات در كودكان چندقلو مشاهده شد، بنابراین آموزش در كل مادران و به خصوص در مورد مادران چندقلوزا باید مورد توجه خاص قرار گیرد.

Title: بررسي ميزان و نوع آلودگي ميکروبي تلفن‌هاي همراه پرسنل درماني شاغل در بيمارستان شهيد بهشتي همدان

http://hsr.mui.ac.ir/index.php/jhsr/article/view/533

مقدمه: تلفن‌هاي همراه توسط پرسنل بيمارستان به طور گسترده‌اي مورد استفاده قرار مي‌گيرند. مطالعه حاضر ميزان بالقوه رشد پاتوژن‌هاي باکتريايي بر روي تلفن‌هاي همراه پرسنل شاغل در بيمارستان شهيد بهشتي شهر همدان را مورد بررسي قرار داده است. روش‌ها: پرسشنامه‌اي جهت بررسي ميزان توجه کارکنان به پاکسازي تلفن‌هاي همراه تهيه شد. از 48 تلفن همراه متعلق به پزشکان و ساير کارکنان شاغل جهت ارزيابي امکان رشد باکتري‌ها، کشت تهيه گرديد و براي نمونه‌برداري، از سواپ استريل مرطوب با مواد مغذي استفاده شد. کشت در تمام جهات جلو، عقب و اطراف تلفن همراه با دقت انجام شد. یافته‌ها: طبق نتايج، 25/56 درصد از تلفن‌هاي همراه فوق آلوده بودند، پس مي‌توان نتيجه گرفت که تلفن‌هاي همراه مي‌توانند به عنوان منبع بالقوه عفونت بيمارستاني باشند. باکتري‌هاي مشاهده شده به ترتيب فراواني، استافيلوکوک کوآگولاز منفي (25/56 درصد)، گونه‌هاي باسيلوس (25/6 درصد) و اشرشياکلي (17/4 درصد) مي‌باشند. نتیجه‌گیری: با توجه به اهميت شستن دست در بيمارستان‌ها و ميزان بالاي عفونت‌هاي بيمارستاني مرتبط با آن، تنها 6 درصد از پرسنل به طور مرتب تلفن همراه خود را با محلول‌هاي ضد عفوني تميز مي‌کردند. بنابراين پاک‌سازي متناوب گوشي‌هاي همراه بايد مدنظر قرار گيرد.

Title: بررسی علل مرگ و میر مادران باردار، ناشي از زايمان در مناطق شهری و روستایی استان ايلام در سال‌های 89-1380

http://hsr.mui.ac.ir/index.php/jhsr/article/view/462

مقدمه: با وقوع مرگ مادران باردار بنیان خانواده و سلامت فرزندان در معرض خطر قرار می‌گیرد. شناخت عوامل مؤثر بر این شاخص مهم، ما را در اتخاذ راهکارهایی جهت پیش‌گیری از بروز مرگ‌های مشابه و بهبود آن، یاری می‌نماید. هدف این پژوهش، تعیین علل مرگ و میر مادران ناشی از زایمان در استان ایلام طی سال‌های 89-1380 بود. روش‌ها: در این پژوهش توصیفی- تحلیلی و گذشته‌نگر، تمام مادران فوت شده به تعداد 25 نفر و گروه مادران زنده بعد از بارداری به تعداد 388 نفر در سال‌های مذکور با استفاده از نمونه‌گیری چند مرحله‌ای انتخاب شدند. سپس اطلاعات آن‌ها از پرونده خانوار گردآوری گردید و با استفاده از نرم‌افزار 16 SPSSو آزمون‌های 2χ، t2-test و Logistic regression مورد تحلیل قرار گرفت. یافته‌ها: شایع‌ترین علت مرگ مادران خونریزی بود و بیش‌ترین درصد مرگ‌ها در 48 ساعت اول پس از زایمان اتفاق افتاده بود. ارتباط معنی‌داری بین مرگ مادران باردار استان و متغیرهای سواد، کیفیت مراقبت دوران بارداری، بارداری پرخطر، ابتلا به بیماری زمینه‌ای و تعداد بارداری وجود داشت. همچنین رابطه معنی‌دار مستقیم بین ابتلای مادران باردار به بیماری زمینه‌ای و بارداری پرخطر و رابطه معکوس در برخورداری مادران باردار از مراقبت‌های دوران بارداری و مرگ مادران به دست آمد. نتیجه‌گیری: افزایش پوشش و کیفیت مراقبت پیش از بارداری، حین بارداری و پس از زایمان؛ ارتقای کیفیت مشاوره تنظیم خانواده و بهبود مهارت و آگاهی کادر پزشکی و مامایی در زمینه مراقبت‌های پس از زایمان از مهم‌ترین اقدامات مؤثر در کاهش مرگ و میر مادران در استان خواهد بود.

Title: بررسی ارتباط برنامه کاری و خستگی در پرسنل مرکز فوريت‌هاي پزشكي شهر اصفهان به روش استاندارد CIS20Rدر سال 1389*

http://hsr.mui.ac.ir/index.php/jhsr/article/view/460

مقدمه: خستگی یکی از علایم شایع تظاهر کننده در حیطه مراقبت‌های اولیه است که بر انجام کار، زندگی خانوادگی و روابط اجتماعی اثرات منفی می‌گذارد. این مطالعه با هدف تعیین ارتباط برنامه کاری و خستگی پرسنل مرکز فوريت‌هاي پزشكي شهر اصفهان به روش استاندارد  CIS20Rدر سال 1389 انجام شد. روش‌ها: این مطالعه از نوع مقطعی بود که بر روی 72 نفر از پرسنل مرکز فوريت‌هاي پزشكي شهر اصفهان انجام شد. برای سنجش برنامه کاری از یک پرسش‌نامه 23 سؤالی و همچنین برای اندازه‌گیری خستگی از پرسش‌نامه CIS20R که شامل 20 سؤال بود، استفاده شد. تجزیه و تحلیل داده‌ها با استفاده از آزمون آماری رگرسیون توسط نرم‌افزار 16SPSS انجام گردید. یافته‌‌ها: نتایج نشان داد که بین برنامه کاری و خستگی ارتباط معنی‌داری وجود دارد (001/0 > P). به طوری که از بین فاکتورهای برنامه کاری، رضایت از طول ساعات کاری، وقفه‌های استراحت و نظم کاری بر روی خستگی مؤثر بودند و فاکتور قابلیت پیش‌بینی بر روی خستگی مؤثر نبود. همچنین، نتایج نشان داد که برنامه کاری به طور عمده بر روی خستگی ذهنی مؤثر است. بدین صورت که ارتباط معنی‌داری بین رضایت از طول ساعات کاری، وقفه‌های استراحت و نظم کاری با خستگی ذهنی به دست آمد. نتیجه‌گیری: با افزایش رضایت از طول ساعت کاری، وقفه‌های استراحتی و همچنین افزایش نظم کاری، می‌توان میزان خستگی پرسنل مرکز فوريت‌هاي پزشكي را کاهش داد.
